# Bsx Is Essential for Differentiation of Multiple Neuromodulatory Cell Populations in the Secondary Prosencephalon

**DOI:** 10.3389/fnins.2020.00525

**Published:** 2020-06-03

**Authors:** Theresa Schredelseker, Florian Veit, Richard I. Dorsky, Wolfgang Driever

**Affiliations:** ^1^Developmental Biology, Institute Biology 1, Faculty of Biology, Albert Ludwig University of Freiburg, Freiburg im Breisgau, Germany; ^2^BIOSS Centre for Biological Signalling Studies, University of Freiburg, Freiburg im Breisgau, Germany; ^3^CIBSS – Centre for Integrative Biological Signalling Studies, University of Freiburg, Freiburg im Breisgau, Germany; ^4^Department of Neurobiology & Anatomy, University of Utah, Salt Lake City, UT, United States

**Keywords:** neuromodulators, evolutionary conservation, brain-specific homeobox (bsx), zebrafish, basal hypothalamus, neuropeptides, aminergic neurons, nitrergic neurons

## Abstract

The hypothalamus is characterized by great neuronal diversity, with many neuropeptides and other neuromodulators being expressed within its multiple anatomical domains. The regulatory networks directing hypothalamic development have been studied in detail, but, for many neuron types, control of differentiation is still not understood. The highly conserved Brain-specific homeobox (Bsx) transcription factor has previously been described in regulating *Agrp* and *Npy* expression in the hypothalamic arcuate nucleus (ARC) in mice. While *Bsx* is expressed in many more subregions of both tuberal and mamillary hypothalamus, the functions therein are not known. Using genetic analyses in zebrafish, we show that most *bsx* expression domains are dependent on Nkx2.1 and Nkx2.4 homeodomain transcription factors, while a subset depends on Otp. We show that the anatomical pattern of the ventral forebrain appears normal in *bsx* mutants, but that Bsx is necessary for the expression of many neuropeptide encoding genes, including *agrp, penka*, *vip*, *trh*, *npb*, and *nts*, in distinct hypothalamic anatomical domains. We also found Bsx to be critical for normal expression of two Crh family members, *crhb* and *uts1*, as well as *crhbp*, in the hypothalamus and the telencephalic septal region. Furthermore, we demonstrate a crucial role for Bsx in serotonergic, histaminergic and nitrergic neuron development in the hypothalamus. We conclude that Bsx is critical for the terminal differentiation of multiple neuromodulatory cell types in the forebrain.

## Introduction

Together with the pituitary gland, the hypothalamus represents the main neuroendocrine center of the body, regulating core physiological parameters such as growth, body temperature, fluid balance, feeding and energy expenditure, stress and arousal, fatigue and sleep, maternal bonding, and lactation. This functional diversity is reflected by the cellular heterogeneity of the hypothalamus, which consists of multiple distinct nuclei harboring a great variety of cell types (Bedont et al., 2015). Beyond truly neuroendocrine cells secreting neurohormones, a great variety of neuromodulators are expressed in the hypothalamus (Romanov et al., 2019). These neuromodulators influence the response behavior of numerous neural circuits, both within and beyond the hypothalamus (Lee and Dan, 2012; Nadim and Bucher, 2014). Biochemically, neuromodulators range from small molecules, such as nitric oxide or monoamines, to peptides, with more than 100 different neuropeptides being known in the human brain (Wang et al., 2015; Russo, 2017).

Thus, the hypothalamus represents an excellent model to study how extraordinary cellular heterogeneity arises from a limited number of progenitor cells. Previous work already suggested that multiple signaling pathways and a highly combinatorial network of transcription factors are required to generate the great diversity of neuromodulatory cell types in the hypothalamus (reviewed in Bedont et al., 2015; Xie and Dorsky, 2017; Alvarez-Bolado, 2019). Deciphering these signaling pathways and transcription factors will deepen our understanding of how this complex brain structure develops, and ultimately may facilitate the establishment of *in vitro* differentiation protocols for use in regenerative approaches to treat neuroendocrine disorders (Suga, 2019). Extensive evolutionary conservation of hypothalamus anatomy, development, and function facilitates comparisons between different animal models from fish to mammals (Löhr and Hammerschmidt, 2011; Machluf et al., 2011; Dominguez et al., 2015; Puelles and Rubenstein, 2015; Santos-Duran et al., 2015; Xie and Dorsky, 2017; Alié et al., 2018; Schredelseker and Driever, 2020).

The highly conserved Brain-specific Homeobox (Bsx) transcription factor has first been reported in *Drosophila* (Jones and McGinnis, 1993). Bsx expression in several subregions of the hypothalamus, the pineal gland, and the telencephalic septum (TelSep) has been described for mice (Cremona et al., 2004) and zebrafish (Schredelseker and Driever, 2018). While recently Bsx functions in the development of the epithalamus have been elucidated (D’Autilia et al., 2010; Schredelseker and Driever, 2018; Mano et al., 2019), no data exist on the role of Bsx in other forebrain regions of teleosts.

In the mouse ARC, *Bsx* has been shown to be coexpressed with *Agouti-related neuropeptide* (*Agrp*) and *Neuropeptide Y* (*Npy*) (Sakkou et al., 2007). Moreover, *Agrp* and *Npy* expression has been found to be strongly reduced during mouse embryonic development in *Bsx* mutants (Sakkou et al., 2007). Follow-up studies showed that upon activation by Ghrelin (Nogueiras et al., 2008) Bsx directly binds the promoter regions of *Agrp* and *Npy* (Lee et al., 2013). Bsx has been discussed nearly exclusively as a regulator of orexigenic peptide expression in the ARC (Burbridge et al., 2016; Alvarez-Bolado, 2019). Bsx functions beyond the regulation of orexigenic factors in cells of the melanocortin system have been scarcely explored, but lactation deficiencies in *bsx* mutant mice have been reported (McArthur and Ohtoshi, 2007). Given the much broader expression domains of *Bsx*, however, we hypothesized that Bsx has additional functions in the development of other cell types in the hypothalamus. Here, we assessed Bsx functions in the secondary prosencephalon with a focus on the bHyp, where *bsx* is broadly expressed in domains that we recently characterized in detail (Schredelseker and Driever, 2020). We identified transcription factors that regulate the expression of *bsx* in the hypothalamus. In *bsx* mutant embryos, we found patterning in the secondary prosencephalon to be normal. To identify Bsx roles in neuronal differentiation, we focused on peptidergic and aminergic neuromodulators. Comparing wildtype and *bsx* mutant zebrafish embryos, we analyzed the expression of genes encoding zebrafish homologs of the neuropeptides assessed by Díaz et al. (2014), with the exception of *oxytocin* (*oxt*), which in the embryonic hypothalamus of both zebrafish and mouse is only expressed in alar regions (Eaton and Glasgow, 2007; Díaz et al., 2014), where *bsx* is not expressed. We extended our analysis to additional markers for peptidergic, nitrergic and monoaminergic neurons. For 13 of the 26 markers analyzed, we detected absent or strongly reduced expression in defined bHyp subregions of *bsx* mutant embryos, demonstrating that Bsx exerts functions beyond the specification of orexigenic neurons in the ARC. In addition, we found Bsx to be required for *uts1* expression in the TelSep.

Notably, we found that Bsx functions are not restricted to a single hypothalamic nucleus, and that Bsx is also not selectively required for expression of a particular gene specific to a certain neuromodulatory cell type. Instead, Bsx appears crucial for expression of multiple genes in distinct clusters distributed over several distinct hypothalamic areas, while the same genes are expressed independently of Bsx in other areas. This supports the idea that the development of the numerous neuromodulatory cell types in the hypothalamus is controlled by transcription factors in a highly combinatorial manner. By demonstrating that Bsx is a determinant of a surprisingly large number of hypothalamic and septal neuromodulatory cell populations, we propose to replace the notion of Bsx as a transcriptional regulator in a single neuron type by a model that presents Bsx as a major developmental factor in many neuromodulatory cell types within and beyond the hypothalamus. Bsx is a crucial component of a so far not fully understood complex combinatorial code for neuromodulatory neuron differentiation.

## Results

### *bsx* Expression Is Differentially Regulated by Homeobox Transcription Factors in Different Regions of the Secondary Prosencephalon

**FIGURE 1:**
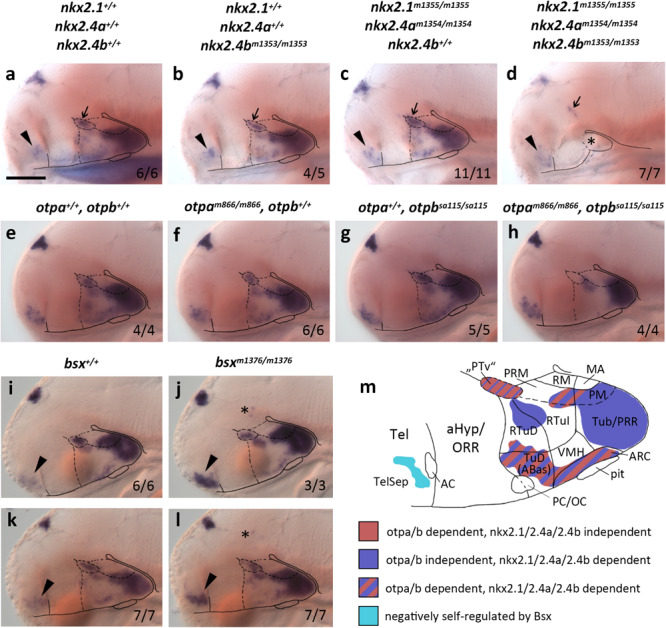
*bsx* expression depends on Nkx2.1, Nkx2.4, and Otp transcription factors. **(a–l)** Lateral views of wildtype and mutant (genotypes as indicated above panels) 4 dpf **(a–d)**, 3 dpf **(e–h,k,l)** or 2 dpf **(i,j)** embryos, stained by *in situ* hybridization for *bsx* expression. All images are minimum intensity projections of 50 brightfield focal planes (distance 1 μm). Anterior is to the left. Scale bar **(a–l)**: 100 μm. The ratio of observed phenotype and total analyzed embryos per genotype [*n* (phenotype)/*n* (total)] is indicated in right bottom corner of each panel. Arrowheads indicates *bsx* expression in the telencephalic septal region; small arrows indicate *bsx* expression which might correspond to “PTv” region; asterisk in **(d)** indicates tissue which might correspond to residual basal hypothalamus (bHyp) but which was not confirmed through marker gene expression in *nkx2.1*, *nkx2.4a*, *nkx2.4b* triple mutants; asterisks in **(j,l)** indicate ectopic *bsx* expressing cells. **(m)** Schematic drawing of a lateral view of *bsx* expressing forebrain regions of a zebrafish embryo 3–4 dpf illustrating which of the *bsx* expression domains depend on Otp or Nkx2.1 and Nkx2.4 transcription factors as indicated by color code. Anterior at left. For abbreviations see list.

**FIGURE 2:**
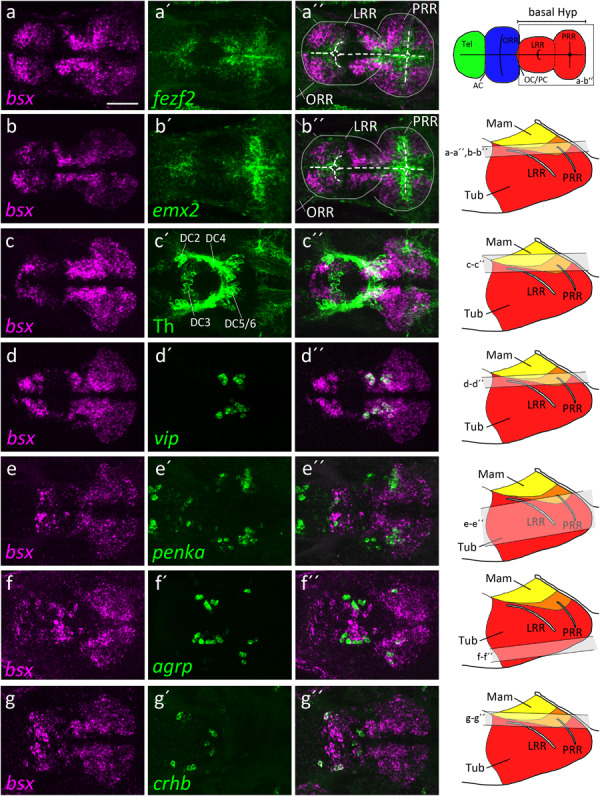
*bsx* expression within the bHyp in relation to expression of neural progenitor markers and neuropeptidergic genes. Dorsal view of confocal sections of zebrafish embryos at 2 dpf **(a–b′′)**, 3 dpf **(c–e′′)** or 4 dpf **(f–g′′)** after double-fluorescent whole-mount *in situ* hybridization using probes as indicated. Maximum intensity *Z*-projections of 30 **(a–b′′,d–d′′)**, 40 **(f–g′′)**, 50 **(c–c′′)**, or 70 **(e–e′′)** single confocal planes (1 μm steps) are shown. Schematics on the right show lateral view of the bHyp for 2–4 dpf embryos indicating which planes were selected for *Z*-projections in the panels indicated. Expression of *bsx* is detected further away from the ventricle [white dashed line in **(a–b′′**)] than expression of neuronal progenitor markers **(a–b′′)**. *bsx* expression colocalizes in neurons expressing Th [anti-Th immunostain; **(c–c′′**)] or neuropeptidergic transcripts **(d–g′′)**. Scale bar in **(a)** for all images: 50 μm. Schematics in the top right represents a model of the zebrafish forebrain highlighting the ventricular recesses. Anterior at left. For abbreviations see list.

While *bsx* expression domains in the bHyp have recently been characterized in detail (Schredelseker and Driever, 2020), no data exist on the upstream regulation of *bsx* expression in the hypothalamus. Severe hypoplasia and deformities in the bHyp were described for *Nkx2.1* mutant mice (Kimura et al., 1996) and critical functions of Nkx-homeodomain factors Nkx2.1, Nkx2.4a, and Nkx2.4b were revealed in zebrafish hypothalamus development (Manoli and Driever, 2014). To asses if hypothalamic *bsx* expression depends on the activity of early acting Nkx-homeodomain transcription factors, we used TALENs to generate loss-of-function alleles for *nkx2.1*, *nkx2.4a* and *nkx2.4b* (Supplementary Figure S1). We analyzed *bsx* expression in single and compound mutants by *in situ* hybridization, and found it to be lost in *nkx2.1*, *nkx2.4a*, and *nkx2.4b* triple mutants (here abbreviated *nkx2.1/2.4a/b*) in all hypothalamic areas of embryos 4 dpf (Figures 1a–d). However, expression of *bsx* is preserved in *nkx2.1/2.4a/b* triple mutants in two ventral forebrain areas. The first one might correspond to an expression domain which in wildtype embryos is located at the border between the alar and basal plate at the rostrocaudal level of the border between diencephalon and secondary prosencephalon (Figures 1a–d, arrow), where *bsx* is expressed in close proximity to the *th* expressing dopaminergic diencephalic cluster 2 (DC2) cells in the “ventral posterior tuberculum” [“PTv”; Figures 2c–c′′ and (Schredelseker and Driever, 2020)]. However, this putative “PTv” domain seem to be reduced in all triple mutants analyzed, suggesting that also this domain is not fully independent of Nkx2.1/2.4a/b factors. Second, *bsx* expression in the TelSep region, which in mice is fused at the midline upon Nkx2.1 loss (Kimura et al., 1996), is normal in *nkx2.1/2.4a/b* triple mutants (Figures 1a–d, arrowhead).

Single *nkx2.4b* mutants or *nkx2.1*, *nkx2.4a* double mutants show reduced expression of *bsx* in the bHyp (Figures 1b,c), which is, however, not nearly as severe as in the triple mutants (Figure 1d). These data are consistent with previous studies showing partial redundancy of Nkx2.1 and Nkx2.4 transcription factors in hypothalamus patterning (Manoli and Driever, 2014). As expected, *bsx* expression in the pineal complex is unaffected in *nkx2.1/2.4a/b* triple mutants (Figures 1a–d).

We recently showed that *bsx* is coexpressed with *orthopedia homeobox a* (*otpa*) in mamillary regions as well as in the terminal tuberal hypothalamus (Schredelseker and Driever, 2020). The two paralogs *otpa* and *otpb* function partially redundantly in the ventral forebrain (Ryu et al., 2007; Fernandes et al., 2013). For *Otp* mutant mice, reduced *Bsx* expression has been reported only for the ARC (Lee et al., 2018). In zebrafish embryos, however, we found *bsx* expression to be lost in *otpa*/*otpb* double mutants not only in the ARC but also in all other regions in which *bsx* colocalizes with *otpa* (3 dpf; Figures 1e–h and Supplementary Figure S2). We concluded that in all *bsx*^+^/*otpa*^+^ regions, i.e., the “PTv”, parts of the Mam as well as the ARC and the dorsotuberal/anterobasal (TuD/ABas) region, *bsx* expression is strictly dependent on Otp transcription factors. To investigate whether Otpa is also sufficient to induce ectopic *bsx* expression, we assessed *Tg(hsp70l:otpa-ires-egfp-caax)* embryos, which, upon heat shock, express *otpa* broadly throughout the brain (Supplementary Figures S3A,B). However, *bsx* expression was normal in embryos 2 dpf even after three waves of *otpa* overexpression induced by heat shocks performed at 12, 24, and 30 hpf (Supplementary Figures S3C,D), indicating that Otpa is not sufficient to activate ectopic *bsx* expression.

**FIGURE 3:**
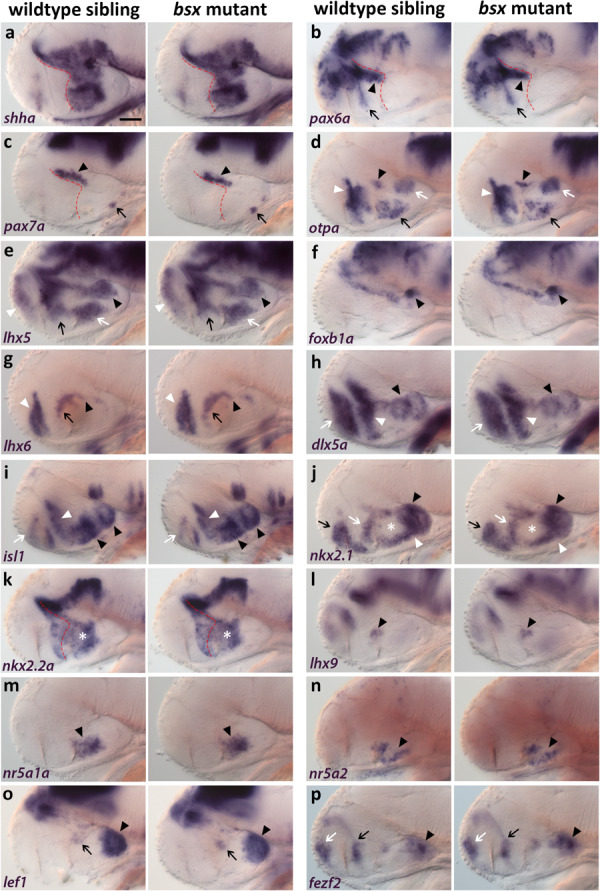
Forebrain patterning is normal in *bsx* mutants. **(a–p)** Lateral views of 2 dpf zebrafish embryo heads stained by *in situ* hybridization using probes as indicated. No differences in expression of any marker was observed between wildtype and *bsx* mutant embryos. All images are minimum intensity projections of 40 brightfield focal planes (distance 1 μm). Anterior is to the left. Scale bar in **(a)** for all images: 50 μm. Anterior at left. Number *n* of embryos analyzed (wildtype) = 3 **(a,e,h,i)**; 5 **(b,m)**; 6 **(c,p)**; 10 **(d)**; 9 **(f,n)**; 14 **(g)**; 8 **(j,k,o)**; 12 **(l)**. *n* (*bsx* mutants) = 3 **(a)**; 11 **(b,g)**; 4 **(c,j)**; 12 **(d)**; 5 **(e,l,o)**; 9 **(f,n,p)**; 8 **(h,i)**; 10 **(k)**; 6 **(m)**.

We further assessed *bsx* expression in *bsx* mutants (Schredelseker and Driever, 2018) to investigate potential autoregulative Bsx functions. We found *bsx* to be expressed normally in the pineal complex and hypothalamus but to be upregulated in the TelSep area in *bsx* mutant embryos (2 dpf: Figures 1i,j; 3 dpf: Figures 1k,l), suggesting negative autoregulation in this area. We also found few dispersed *bsx* expressing cells around the di-mesencephalic boundary in all *bsx* mutant embryos (Figures 1j,l, asterisk) but never in wildtypes. We summarized the upstream regulation of *bsx* in different domains of the forebrain in Figure 1m.

### *bsx* Is Expressed in Differentiating Neurons Rather Than in Neural Stem Cells

The hypothalamus, like many brain regions, displays a pronounced centrifugal ventricular to mantle gradient of neurogenesis and cell maturation (Affaticati et al., 2015; Alvarez-Bolado, 2019). By double-fluorescent *in situ* hybridization, we compared *bsx* expression along the apical to basal axis of the hypothalamic neuroepithelium with expression of genes characteristic for proliferating neural stem cells or postmitotic differentiated neurons. Expression of the neural stem and radial glia markers *emx2* and *fezf2* (Cecchi, 2002; Yang et al., 2012; Alvarez-Bolado, 2019) are restricted to areas close to the ventricle, while *bsx* expression was found to be located further away from the ventricle in mantle zones (Figures 2a–b′′).

We then compared *bsx* expression to markers of differentiated neurons. We detected *bsx* expression in dopaminergic DC5 and 6 cells in the mamillary hypothalamus (Figures 2c–c′′). In the mamillary hypothalamus, we also found cells expressing both *bsx* and *vasoactive intestinal peptide* (*vip*) (Figures 2d–d′′). The location of those *vip*^+^ cells within the *otpa*^+^ positive parts of the mamillary hypothalamus has been shown by others (Wolf and Ryu, 2013). We recently found medial regions of this brain area to correspond to mamillary hypothalamus, while more lateral regions express markers characteristic for the tuberal hypothalamus (Schredelseker and Driever, 2020). Within the tuberal hypothalamus, lateral to the *vip* expressing cells, as well as in the TuD/ABas region, we found cells expressing both *bsx* and the endogenous opioid preproprotein *proenkephalin a* (*penka*) (Figures 2e–e′′). In an adjacent region of the terminal tuberal hypothalamus, corresponding to the ARC region (Schredelseker and Driever, 2020), we also detected *agrp* cells within the *bsx* expressing territories (Figures 2f–f′′). Notably, in the border region between alar and basal plate at the rostrocaudal level of the border between hypothalamus and diencephalon, in which also the *th* expressing dopaminergic DC2 cluster is located, we found *crhb* cells to express *bsx* (Figures 2g–g′′). Taken together, these data suggest that *bsx* is expressed in mantle postmitotic differentiating neurons rather than in ventricular neural stem or progenitor cells.

### Forebrain Patterning Is Normal in *bsx* Mutants

To investigate a potential role of Bsx in hypothalamus patterning, we compared *bsx* mutants with wildtype siblings for gene expression patterns which define specific progenitor domains in the embryonic hypothalamus. *sonic hedgehog a* (*shha*) expression in the secondary prosencephalon marks the alar-basal boundary [(Puelles et al., 2012); Figure 3a, red dashed line; for regional organization of the hypothalamus see Figure 1m]. Expression of *shha* being unaffected in *bsx* mutants suggests a normal course of the alar-basal boundary in *bsx* mutants (Figure 3a). Expression of *pax6a* and *pax7a* is unaffected in *bsx* mutants compared to wildtype (Figures 3b,c, arrowhead), indicating regular formation of alar and basal parts of prosomer 3, respectively. Unchanged expression of *pax7a* in the pituitary further indicates proper formation of the pituitary intermediate lobe (Figure 3c, arrow). *pax6a* in the telencephalon and in the progenitor cells lining the optic recess (Figure 3b, arrow) is also expressed identically to wildtype siblings in *bsx* mutants.

*otpa* expression is normal in the “PTv” (Figure 3d, black arrowhead), perimamillary (PM) region (Figure 3d, white arrow), TuD/ABas area (Figure 3d, black arrow) as well as in the aHyp/ORR (Figure 3d, white arrowhead). Similarly, *lhx5* expression is unchanged in mamillary regions (Figure 3e, black arrowhead), TuD/ABas (Figure 3e, white arrow), aHyp/ORR (Figure 3e, black arrow) and the telencephalon (Figure 3e, white arrowhead). Normal *foxb1a* expression indicates the Mam to develop properly (Figure 3f, black arrowhead). *lhx6* expression is unchanged in the pallidum (Figure 3g, white arrowhead), in the aHyp/ORR (Figure 3g, black arrow), as well as in the bHyp (Figure 3g, black arrowhead) where in mouse it has been demonstrated to selectively mark RTuV/TuV (Puelles et al., 2012).

Also other tuberal regions appear to be well developed in *bsx* mutants, as judged from unaltered expression of *dlx5a* (Figure 3h, black arrowhead), which marks all tuberal regions except the core region of the ventromedial hypothalamus (VMH) (Puelles et al., 2012; Morales-Delgado et al., 2014), and of *isl1* (Figure 3i, black arrowheads), which marks all tuberal regions (Puelles et al., 2012). Expression of *dlx5a* and *isl1* is also unchanged in the telencephalon (Figures 3h,i, white arrow) and aHyp/ORR (Figures 3h,i, white arrowhead). *nkx2.1* expression is normal in both mamillary and tuberal regions (Figure 3j, black and white arrowheads, respectively) as well as in the telencephalon and aHyp/ORR (Figure 3j, black and white arrow, respectively). The gap in *nkx2.1* expression in the intermediate and dorsal retrotuberal (RTuI/RTuD) regions (Figure 3j, white asterisk) was observed previously (Rohr et al., 2001; Manoli and Driever, 2014), and is also unchanged in *bsx* mutants. In this *nkx2.1* negative territory, *nkx2.2a* is expressed (Figure 3k, white asterisk). *nkx2.2a* has been described to be expressed in both the liminal and subliminal band along the alar-basal boundary (Puelles et al., 2012; Figure 3k, red dashed line), and is expressed identically to wildtype in *bsx* mutants. *lhx9* expression indicates normal development of the RTuD/PBas region (Puelles et al., 2012; Figure 3l, black arrowhead). *nr5a1a* and *nr5a2* are expressed in equal patterns in wildtype and *bsx* mutant embryos, suggesting proper formation of TuD/ABas, VMH and the ARC region (Kurrasch et al., 2007; Bedont et al., 2015; Xie and Dorsky, 2017) (Figures 3m,n, black arrowheads). The PRR also appears to develop orderly, as observed through *lef1* and *fezf2* expression (Levkowitz et al., 2003; Wang et al., 2012; Figures 3o,p, black arrowheads). A small expression domain of *lef1* in the RTu region (Figure 3o, black arrow), which has previously been described in mice (Ferran et al., 2015), also appears to be unchanged in *bsx* mutants. *fezf2* expression is normal along the optic recess and in the telencephalon (Figure 3p, black and white arrow, respectively). In conclusion, we could not detect any difference in patterning of the secondary prosencephalon between wildtypes and *bsx* mutants.

### Expression of *agrp* and *penka*, but Not *pomca* or *npy*, in Terminal Regions of the Tuberal Hypothalamus Depends on Bsx

**FIGURE 4:**
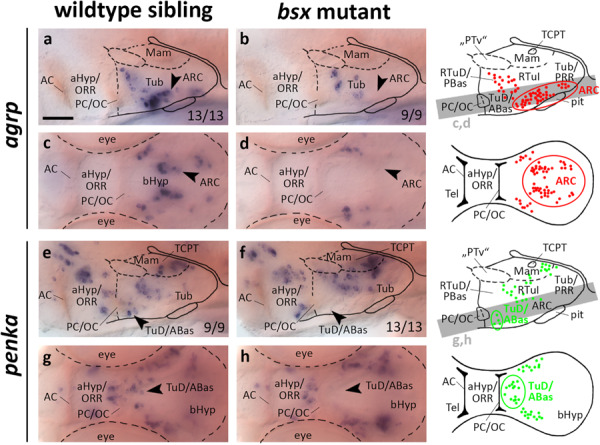
*agrp* and *penka* expression domains in the terminal tuberal hypothalamus are lost in *bsx* mutant embryos. Lateral **(a,b,e,f)** and dorsal views **(c,d,g,h)** of the ventral forebrain in 3 dpf embryos stained by *in situ* hybridization using probes as indicated. All images are minimum intensity projections of 40 brightfield focal planes (distance 1 μm). Scale bar in **(a)** for all images: 50 μm. Anterior at left. Schematics to the right show location of *agrp* (red) or *penka* (green) expressing cells as observed by stainings and indicate the focal planes which are shown in dorsal view pictures and schematics. Cells with Bsx-dependent expression are circled. Numbers *n* (phenotype shown)/*n* (total analyzed) as indicated.

In the mouse ARC, Bsx has been shown to regulate expression of *Agrp* and *Npy*, but not *Pomc* (Sakkou et al., 2007). We found *agrp* expression in the putative ARC area to be lost in *bsx* mutants at 3 dpf (Figures 4a–d, arrowheads) and 4 dpf (Supplementary Figures S4A–D, arrowheads), while *pomca* is expressed normally at both 3 dpf (Supplementary Figure S8I) and 4 dpf (Supplementary Figure S9I), indicating that Bsx functions are conserved in zebrafish. We observed a few dispersed *agrp* expressing cells in peduncular RTu regions which are unaffected by loss of Bsx (Figures 4a–d). We do not know whether these cells represent a distinct cluster which remains anatomically and potentially functionally disjunct from the ARC population, whether those cells are migratory, and/or whether those cells express *agrp* only transiently during embryonic development. Notably, *npy* is not expressed in the ARC area in zebrafish larvae, and we could not detect altered *npy* expression in any brain region of *bsx* mutant embryos at 3 dpf (Supplementary Figure S8H) or 4 dpf (Supplementary Figure S9H).

Given that *bsx*, in mice and in teleosts, is expressed not only in the ARC, but also in multiple other regions of the bHyp (Cremona et al., 2004; Schredelseker and Driever, 2020; Figures 1, 2), we analyzed several other neuropeptidergic cell populations therein. Since we demonstrated several clusters of *penka* expressing neurons to be located within the *bsx* expression domain in both the LRR and PRR (Figures 2e–e′′), we analyzed *penka* expression in *bsx* mutant embryos. We found *penka* expression in one cell cluster within the LRR to be lost in *bsx* mutants at 3 dpf (Figures 4e–h, arrowheads) and 4 dpf (Supplementary Figures S4E–H, arrowheads). In prosomeric anatomical terms this cluster is located in the terminal TuD/ABas region (Schredelseker and Driever, 2020). We concluded that in addition to the role of Bsx on *agrp* neuron differentiation, which has been shown previously in mice (Sakkou et al., 2007), Bsx also functions in differentiation of *penka* neurons in the terminal tuberal hypothalamus in zebrafish.

### Expression of *vip*, *npb*, and *trh* in Subregions of the Mamillary Region Is Dependent on Bsx

**FIGURE 5:**
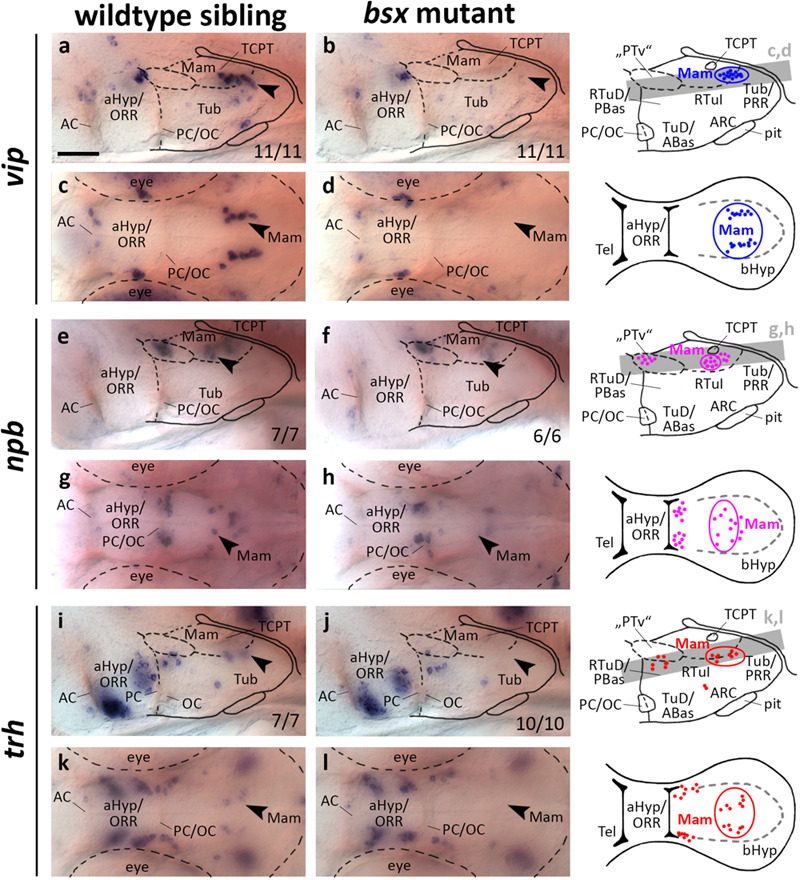
*vip, npb*, and *trh* expression domains in mamillary hypothalamic regions are lost in *bsx* mutant embryos. Lateral **(a,b,e,f,i,j)** and dorsal views **(c,d,g,h,k,l)** of the ventral forebrain in 3 dpf embryos stained by *in situ* hybridization using probes as indicated. All images are minimum intensity projections of 40 brightfield focal planes (distance 1 μm). Scale bar in **(a)** for all images: 50 μm. Anterior at left. Schematics to the right show location of *vip* (blue), *npb* (magenta), or *trh* (red) expressing cells as observed by stainings and indicate the focal planes which are shown in dorsal view pictures and schematics. Cells with Bsx-dependent expression are circled. Numbers *n* (phenotype shown)/*n* (total analyzed) as indicated.

In mice, *vip* expressing cells originate in the aHyp (Díaz et al., 2014) and play a crucial role in circadian rhythm regulation (Maywood et al., 2006). In contrast, during zebrafish development *vip* is expressed not only in the aHyp/ORR but also in mamillary regions of the bHyp, where *vip* expression is dependent on Otp transcription factors (Wolf and Ryu, 2013). We found the mamillary *vip* cluster to be absent in *bsx* mutants at 3 dpf (Figures 5a–d, arrowheads) and 4 dpf (Supplementary Figures S5A–D, arrowheads). Taken together with the observation that *bsx* expression in the Mam is reduced in *otpa*/*otpb* double mutants (Figures 1e–h), we hypothesized that Bsx acts downstream of Otp factors in the differentiation of *vip* neurons.

In a neighboring domain within the Mam, in close proximity to the tract of the commissure of the caudal tuberculum (TCPT; Wilson et al., 1990), we found expression of *npb* to be strongly reduced upon Bsx loss both at 3 dpf (Figures 5e–h, arrowheads) and 4 dpf (Supplementary Figures S5E–H, arrowheads). In mouse, *Npb* is expressed in multiple nuclei within the telencephalon, aHyp and tuberal hypothalamus (Takenoya et al., 2013), and has been shown to be involved in pain processing, circadian rhythm, sleep/wake regulation, and feeding behavior (Dvorakova, 2018). To our knowledge, thus far, no reports exist on *npb* expression in mamillary regions, or on *npb* functions in zebrafish.

Thyrotropin-releasing hormone (Trh) neurons have been implicated in energy homeostasis, arousal and cold response (Hollenberg, 2008; Hara et al., 2009; Zhang et al., 2018). We found a cluster of *trh* expressing cells that is absent in the Mam of *bsx* mutants at 3 dpf (Figures 5i–l, arrowheads) and 4 dpf (Supplementary Figures S5I–L, arrowheads). We conclude that Bsx is essential for differentiation of neuropeptidergic cell clusters not only within the developing ARC but also in multiple other regions within the bHyp.

### Expression of *hcrt*, *cart4*, *sst1.1*, *pdyn*, *galn*, *avp*, *pmch*, *pmchl, npvf*, and *nmu* Is Normal in *bsx* Mutant Embryos

Of the 16 neuropeptide encoding genes analyzed by Díaz et al. (2014), for 11 expression has been reported to originate in the bHyp in mouse. Except for *growth hormone-related hormone* (*ghrh*), which we could not detect in the zebrafish hypothalamus at 3 or 4 dpf (data not shown), and *oxytocin*, which in zebrafish larvae as in mice is restricted to aHyp regions (Unger and Glasgow, 2003; Díaz et al., 2014), we found zebrafish homologs for all other genes to be expressed in at least few cells within the bHyp. In addition to *npy* and *pomca* which we previously mentioned, we detected the expression of *hypocretin* (*hcrt*), *cocaine- and amphetamine-regulated transcript 4* (*cart4*), *somatostatin 1.1* (*sst1.1*), *prodynorphin* (*pdyn*), *galanin* (*galn*), *arginine vasopressin* (*avp*), *pro-melanin-concentrating hormone* (*pmch*), *pro-melanin-concentrating hormone, like* (*pmchl*), *neuropeptide VF precursor* (*npvf*), and *neuromedin U* (*nmu*) in *bsx* mutants to be similar to wildtype both at 3 dpf (Supplementary Figure S8) and 4 dpf (Supplementary Figure S9). Therefore, many neuropeptide encoding genes are normally expressed in *bsx* mutants, and Bsx appears to be specifically required only for expression of a defined group of neuropeptide precursor genes.

### Bsx Is Required for Normal Development of the Crh System and for *uts1* Expression in the Telencephalic Septal Region

In teleosts, four genes encode the corticotropin-releasing hormone (Crh) family of ligands: *crha*, *crhb*, *uts1*, and *urocortin 3 like* (Cardoso et al., 2016). Their binding to the Crh receptors is additionally regulated through presence and concentration of a Crh binding protein (Crhbp) (Ketchesin et al., 2017). The Crh system is a core component of the stress response acting through the hypothalamic-pituitary-adrenal axis (Denver, 2009), but has also been implicated in energy homeostasis and appetite regulation (Matsuda, 2013).

**FIGURE 6:**
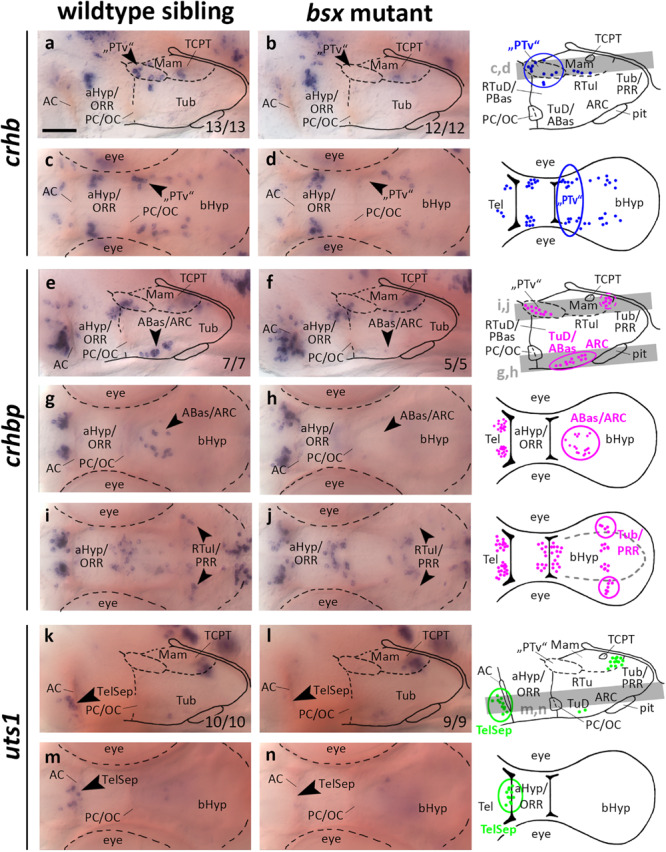
*crhb, crhbp* and *uts1* expression domains in the secondary prosencephalon are lost in *bsx* mutant embryos. Lateral **(a,b,e,f,k,l)** and dorsal views **(c,d,g–j,m,n)** of the ventral forebrain in 3 dpf embryos stained by *in situ* hybridization using probes as indicated. All images are minimum intensity projections of 40 brightfield focal planes (distance 1 μm). Scale bar in **(a)** for all images: 50 μm. Anterior at left. Schematics to the right show location of *crhb* (blue), *crhbp* (magenta), or *uts1* (green) expressing cells as observed by stainings and indicate the focal planes which are shown in dorsal view pictures and schematics. Cells with Bsx-dependent expression are circled. Numbers *n* (phenotype shown)/*n* (total analyzed) as indicated.

In the embryonic zebrafish brain *crhb* expressing cells in the “PTv” region have been found not only to be located in close proximity to the *th* expressing DC2 dopaminergic neurons, but also to be expressed under control of shared transcriptional regulation (Löhr et al., 2009). For instance, “PTv” *crhb* neurons also depend on the Otp transcription factors (Löhr et al., 2009). When we assessed *crhb* expression in the “PTv” region in *bsx* mutants, we found this cell cluster to be absent (Figures 6a–d, arrowheads, and Supplementary Figures S6A–D, arrowheads). Taken together with our observation that *bsx* expression in this region is dependent on Otp (Figures 1e–h), we conclude that Otp may contribute to differentiation of *crhb* neurons in the “PTv” area by activation of *bsx* expression, or that both factors may act in a combinatorial manner in *crhb* differentiation.

*crhbp* in the embryonic zebrafish is expressed in the telencephalon, aHyp/ORR, “PTv” area, and the border region between tuberal and mamillary regions of the bHyp (Figures 6e–j and Supplementary Figures S6E–H, arrowheads). Additionally, a cluster of *crhbp* expressing cells was found in the terminal tuberal hypothalamus reaching from the TuD/ABas region to the ARC (Figures 6e–h, black arrowheads). This cluster was not detected in *bsx* mutants (Figures 6f,h, arrowheads, and Supplementary Figures S6E–H, arrowheads), indicating a differentiation defect of those neurons upon loss of Bsx function. In the border region between mamillary and tuberal regions we found a far laterally located cluster of *crhbp* expressing cells to be absent in *bsx* mutants (Figures 6i,j, arrowheads), while more medially located *crhbp* cells appear to be unaffected. Based on our previous studies (Schredelseker and Driever, 2020), within the PRR we associated the Bsx-dependent lateral *crhbp* cells with tuberal regions and the Bsx-independent cells with mamillary regions.

*urotensin 1* (*uts1*) expression in the zebrafish Mam has been demonstrated to be dependent on Otp transcription factors (Wolf and Ryu, 2013). A member of the mammalian homologs to Urotensin, Urocortin 1, has been detected in the lateral telencephalic septum (Kozicz et al., 1998), which is of pallidal origin in mice (Medina and Abellán, 2012). We detected a few *uts1* expressing cells adjacent to the AC in an area hypothesized to be homologous to the mammalian TelSep region (Schredelseker and Driever, 2020; Figure 6k, arrowhead). We could not detect any *uts1* expressing neurons in this region in *bsx* mutant embryos 3 dpf (Figures 6k–n, arrowheads) or 4 dpf (Supplementary Figures S6I–L, arrowheads). Since we have demonstrated the pallidum to develop normally as inferred from normal expression of *nkx2.1* and *lhx6* (Figure 3), we conclude that Bsx is critical for differentiation of *uts1* neurons, but not for patterning of the pallidal domain.

### Bsx Functions in Differentiation of Monoaminergic Cells in the Posterior Recess Region

For the PRR we recently proposed homology of ventral and dorsolateral parts to tuberal regions, and of dorsomedial parts to mamillary regions, based on gene expression data (Schredelseker and Driever, 2020). Still, the phylogenetic relationship of the PRR remains particularly elusive as most phyla lack a posterior hypothalamic recess (Vernier, 2017; Yamamoto et al., 2017). Cell types within the teleost-specific PRR are well characterized and include many monoaminergic cerebrospinal fluid contacting (CSF-c) cells (Eriksson et al., 1998; Filippi et al., 2010; Xavier et al., 2017; Xie et al., 2017).

**FIGURE 7:**
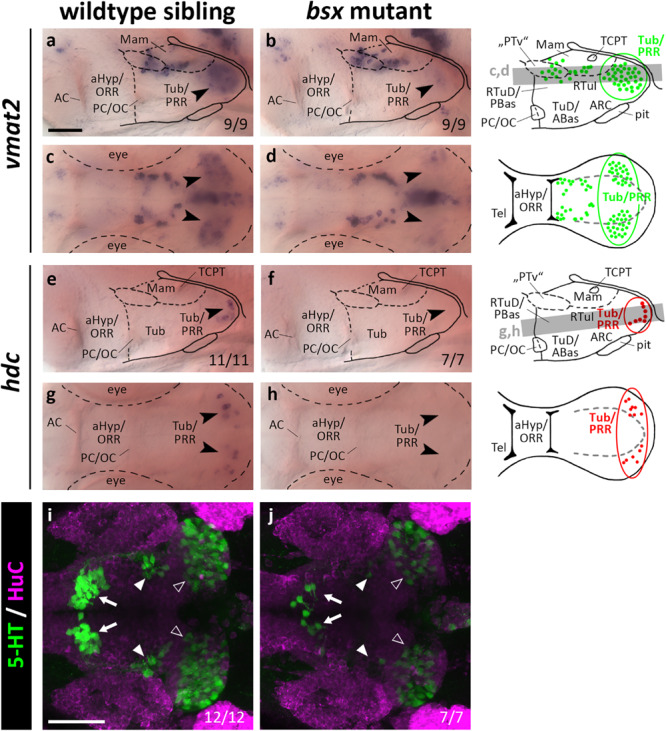
*vmat2* expression, *hdc* expression and 5-HT immunoreactivity in the posterior recess region are reduced or absent in *bsx* mutant embryos. Lateral **(a,b,e,f)** and dorsal views **(c,d,g,h)** of the ventral forebrain in 3 dpf embryos stained by *in situ* hybridization using probes as indicated. **(i,j)** Dorsal view of the ventral forebrain in 3 dpf embryos immunostained using 5-HT (green) and HuC antibody (magenta). Images show minimum intensity projections of 40 brightfield focal planes (**a–h**; 1 μm steps) or maximum intensity projections of 40 confocal planes (**i,j**; 1 μm steps). Scale bars in **(a)** for **(a–h)** and in **(i)** for **(i)** and **(j)**: 50 μm. Anterior at left. Schematics to the right show location of *vmat2* (green) and *hdc* (red) expressing cells as observed in stainings and indicate the focal planes which are shown in dorsal view pictures and schematics. Cells with Bsx-dependent expression are circled. Numbers *n* (phenotype shown)/*n* (total analyzed) as indicated.

Since *bsx* is expressed in the PRR, we aimed to assess the differentiation of monoaminergic cells therein. We made use of *vmat2* as a marker for all monoaminergic cell types, of *hdc* as a specific marker for histaminergic cells, and of *th* as well as its paralog *th2* to label dopaminergic cells. Since *tryptophan hydroxylase* expression is low in hypothalamic serotonergic cells (Bellipanni et al., 2002), we made use of an antibody recognizing a 5-HT/paraformaldehyde conjugate (Bosco et al., 2013). We found the expression of *vmat2* to be strongly reduced in the PRR (Figures 7a–d, arrowheads, and Supplementary Figures S7A–D, arrowheads), indicating that monoaminergic cells develop abnormally in *bsx* mutants. The presence of *hdc* expressing neurons in the PRR supports our hypothesis that parts of the PRR are homologous to the RTuV/TuV territory as defined by the prosomeric model, in which RTuV/TuV has been proposed to be the only source of histaminergic neurons in the brain (Puelles et al., 2012). We detected no *hdc* expressing cells in *bsx* mutants 3 dpf (Figures 7e–h, arrowheads) and 4 dpf (Supplementary Figures S7E–H, arrowheads), and concluded that all histaminergic cells present in the brain at these embryonic stages are strictly Bsx-dependent. We found both the expression of *th1* (DC7; Supplementary Figures S10A–H) and *th2* (Supplementary Figures S10I–L) to be normal in the *bsx* mutant PRR, suggesting dopaminergic cells to develop in a Bsx-independent manner. The *th* expressing cell clusters DC2, 4, 5, and 6 have previously been shown to be dependent on Otp (Ryu et al., 2007). Since we found multiple Otp-dependent expression domains of neuropeptidergic genes also to be affected in *bsx* mutants, we were surprised to find *th* expression in these clusters to be unaffected by Bsx loss (Supplementary Figures S10A–H). This indicates that Otp transcription factors function independently of Bsx in dopaminergic differentiation.

In *bsx* mutants, 5-HT immunoreactivity is strongly reduced not only in the PRR (Figures 7i,j, white outline arrowheads) but also in the other two hypothalamic serotonergic cell clusters (Figures 7i,j, filled white arrowheads and arrows). We conclude that Bsx is crucial for the terminal differentiation of histaminergic and serotonergic, but not dopaminergic cells in the hypothalamus.

### Bsx Is Required for Normal *nts* and *nos1* Expression in the Posterior Recess Region

**FIGURE 8:**
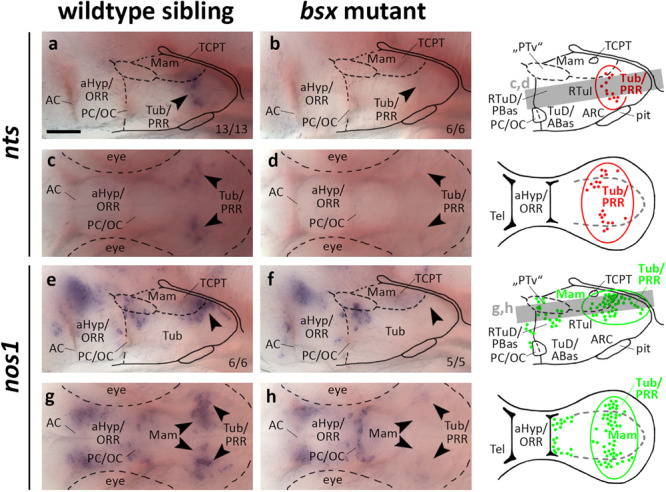
*nts* and *nos1* expression in the posterior recess region is lost in *bsx* mutant embryos. Lateral **(a,b,e,f)** and dorsal views **(c,d,g,h)** of the ventral forebrain in 3 dpf embryos stained by *in situ* hybridization using probes as indicated. All images are minimum intensity projections of 40 brightfield focal planes (distance 1 μm). Scale bar in **(a)** for all images: 50 μm. Anterior at left. Schematics to the right show location of *nts* (red) or *nos1* (green) expressing cells as observed in stainings and indicate the focal planes which are shown in dorsal view pictures and schematics. Cells with Bsx-dependent expression are circled. Numbers *n* (phenotype shown)/*n* (total analyzed) as indicated.

Neurotensin attracted much attention as an important neuromodulator of multiple, and most notably dopaminergic, neuronal circuits (Dobner and Carraway, 2013). We detected *nts* expression in the PRR of wildtype but not *bsx* mutant embryos 3 dpf (Figures 8a–d, arrowheads). At 4 dpf we detected few *nts* expressing cells in the PRR of *bsx* mutants, while wildtype embryos at that stage showed a stronger signal (Supplementary Figures S11A–D, arrowheads).

*nos1* has been demonstrated to be coexpressed in *th* expressing neurons in *Xenopus* (Lopez et al., 2005) and zebrafish embryos (Poon et al., 2003). Functionally, hypothalamic *nos1* has been implicated in energy homeostasis and feeding regulation as well as fertility and reproduction (Leshan et al., 2012; Sutton et al., 2014; Chachlaki et al., 2017). We detected widespread expression of *nos1* in the PRR reaching from medial mamillary regions to lateral Tub regions, which, however, is absent in *bsx* mutants at 3 dpf (Figures 8e–h, arrowheads). At 4 dpf we found few *nos1* expressing cells in the more laterally located tuberal regions of *bsx* mutants while the more medial cells in mamillary regions remained absent (Supplementary Figures S11E–H, arrowheads). Another *nos1* expression domain, consisting of cells spread along the “PTv” and RTuD/PBas region, was unaffected by Bsx loss both at 3 dpf (Figures 8e–h, arrowheads) and 4 dpf (Supplementary Figures S11E–H, arrowheads), indicating that *nos1* expression in the PRR, but not the “PTv” or RTuD/PBas region, strictly depends on Bsx. We conclude that Bsx functions in the differentiation of the nitric oxide and neurotensin neuromodulatory systems in the PRR.

## Discussion

**FIGURE 9:**
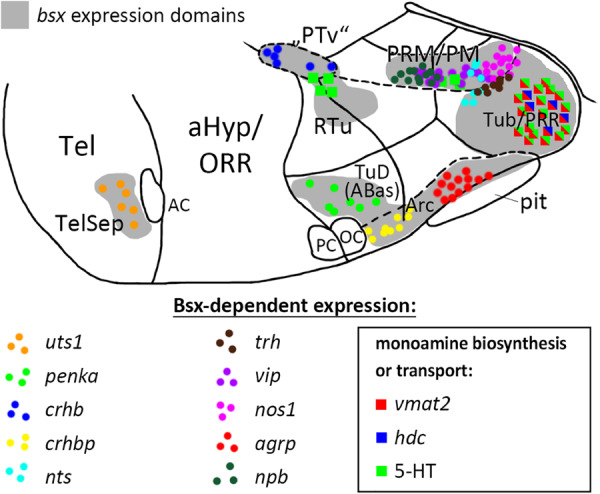
Distribution of Bsx-dependent neuronal cell types. Schematic representation of a sagittal view of the ventral forebrain in 3–4 dpf embryos showing *bsx* expression domains in gray. Only Bsx-dependent neuronal cells are shown and color coded as indicated. The number of symbols reflects the spatial extend of the affected domain but not cell numbers For abbreviations see list. Anterior at left.

In wildtype and *bsx* mutant embryos, we analyzed the expression of different monoamine pathway components and multiple neuropeptidergic precursor genes, for many of which the expression in the zebrafish forebrain has not previously been described in detail. We demonstrate that Bsx is necessary not only for normal expression of a surprisingly large number of peptidergic neuromodulators, but also for the expression of enzymes involved in the synthesis of small neuromodulatory molecules, such as histamine and nitric oxide, and for normal serotonin levels in the hypothalamus (Figure 9). Even though the patterning and gross anatomical structure of the secondary prosencephalon is unchanged in *bsx* mutants, they do not develop the full complement of neuromodulatory molecules in the forebrain, which might have strong implications on physiology and behavior of animals devoid of functional Bsx.

Our data reveal that all *bsx* expression in the basal prosencephalon depends on combined activity of *nkx2.1* and *nkx2.4a/b* genes in zebrafish. Expression of these three genes is maintained during all phases of *bsx* expression studied here (Manoli and Driever, 2014), thus it is possible that Nkx2.1 and 2.4 transcription factors may directly control *bsx* expression. However, embryos devoid of *nkx2.*1 and *nkx2.4a/b* develop severe patterning abnormalities in the hypothalamus with lack of most or all hypothalamic regions in which *bsx* will be expressed (Manoli and Driever, 2014). Therefore, also indirect regulation is possible. Downregulation of *bsx* expression in the ARC upon loss of the Otp transcription factor has recently been shown in mice (Lee et al., 2018), and our data reveal conserved Otp regulation of *bsx* in teleosts. It is unclear how well conserved this gene regulatory network is beyond the ARC, as for *Otp* knock-out mice no reports exist on *Bsx* expression in those areas of the mouse bHyp for which we demonstrate dependency on Otp factors in zebrafish. Other functions of Otp, in contrast, appear to be Bsx-independent, since expression of *th* and *avp* is reduced in *otp* mutants (Ryu et al., 2007; Fernandes et al., 2013), but not in *bsx* mutants.

We recently demonstrated prosomeric organization of the bHyp to be highly conserved in mammalian and teleost embryos (Schredelseker and Driever, 2020). The homology relations of hypothalamic subregions that we proposed in this model are also supported by the expression domains of many neuropeptidergic and aminergic genes that were assessed in the present study. This combined information may also help to understand additional anatomical features. For instance, the location of commissures, visible in stained embryos by mere tissue contrast, is in line with our anatomical analysis and shall be illustrated by one example. We proposed large parts of what was previously suggested to belong to the “PTv” (thus being a basal part of prosomer 3), to actually correspond to mamillary hypothalamic regions (Schredelseker and Driever, 2020). We should thus reconsider the anatomical status (and the name) of the TCPT (Wilson et al., 1990), and, based on its location, hypothesize this commissure to correspond to the retromamillary commissure in mouse (Puelles et al., 2012).

The high degree of conservation in both protein structure and expression pattern of Bsx suggests that many of the herein shown Bsx functions in neuronal differentiation might be conserved in other phyla. However, in demonstrating the loss of monoaminergic markers in the PRR, we also describe Bsx functions which might have undergone substantial evolutionary divergence. The special phylogenetic status of the PRR has been discussed previously (Vernier, 2017; Xavier et al., 2017; Schredelseker and Driever, 2020). The dependence of the monoaminergic CSF-c cells that densely populate the PRR on Bsx might help elucidating the evolutionary relationship of these neurons across vertebrates.

With respect to neuropeptides, we find conservation of *agrp* regulation by Bsx in zebrafish, as reported for mice (Sakkou et al., 2007). As of yet, no reports exist on Bsx functions in other subregions of the developing hypothalamus beyond the ARC. Here, we present Bsx functions in the differentiation of monoaminergic and neuropeptidergic cell types within several subregions of both, the tuberal and mamillary hypothalamus, and the telencephalic septum (summarized in Figure 9, only cell types affected in *bsx* mutants are shown). It should be noted that for all regions in which we found a neuronal differentiation gene not to be expressed in *bsx* mutants, we found other marker genes to be unaffected by Bsx loss. Moreover, for each expressed neuronal differentiation gene affected by Bsx loss in specific regions, we found expression of the same neuronal differentiation gene being not regulated by Bsx in other regions. Together, these findings support the conclusion that the differentiation program for the hypothalamus is regulated in a highly combinatorial manner, and that Bsx functions are context-dependent, rather than Bsx being a specific determinant for a defined cell type. In the case of *penka*, *pmchl, avp*, *vip* and *npb*, we observed, in some embryos, ectopic expression and/or a seemingly denser *in situ* hybridization staining intensity in certain Bsx-independent clusters, which, however, we did not quantify. If confirmed in future studies, this would indicate that Bsx regulates fate switches in the differentiation programs of neuromodulatory cells in the hypothalamus, such that loss of one cell type may correspond to more cells of another type. Bsx function appears to be limited to differentiation rather than patterning mechanisms, as the gross anatomy of the bHyp is unchanged in *bsx* mutants. Notably, Jones and McGinnis previously hypothesized in 1993 that the *Drosophila bsx* homolog *bsh* “is not required for the actual morphology of brain cells, the axonal pathways, etc., but instead regulates expression of neurotransmitters, receptors, channels, synaptic specializations, or other effector molecules that are critical to the physiologic function of particular neural cells” (Jones and McGinnis, 1993).

We were surprised by the strong effects of Bsx loss on histaminergic and serotonergic differentiation in the hypothalamus. Two different Ets domain transcription factors, Pet1 and Etv5b, specify Raphe nucleus and hypothalamic serotonergic neurons, respectively (Hendricks et al., 2003; Bosco et al., 2013). Here, we show that Bsx is an additional component of the transcriptional network that specifies serotonergic differentiation in the hypothalamus, but not the Raphe nucleus. Since in mammals serotonergic somata became restricted to the Raphe, functional characterization of the hypothalamic serotonergic system is sparse. Recent work suggests that the activity of hypothalamic serotonergic cells correlates with hunger states in zebrafish (Wee et al., 2019). The histaminergic system has been well described in zebrafish, but transcriptional regulators of its development have not been reported so far (Chen et al., 2016).

While *bsx* is also expressed in a subset of dopaminergic neurons in the mamillary hypothalamus, the expression of the dopaminergic marker genes *th* and *th2* is normal in *bsx* mutants. DC2, 4, 5, and 6 dopaminergic cells and adjacent neuropeptidergic cell types, including *crhb* expressing neurons, are specified by a shared transcriptional network (Löhr et al., 2009). So far, no transcription factors specific for only one of those two lineages have been found. In demonstrating that *th* expression is unaffected by Bsx loss, while adjacent *crhb* expression is lost, we identify Bsx as a factor that separates those lineages. A model has been put forward that neurons, particularly in the hypothalamus, may frequently express more than one neurotransmitter type, including neuropeptide precursor peptides (Romanov et al., 2019). Therefore, it is tempting to speculate that Bsx may be required in dopaminergic neurons to establish the full complement of neuromodulatory factors which they might express in addition to *th*.

It has recently been suggested that the functional homolog of the *crhb* expressing neurons in the mammalian PVN, which stimulate corticotropes in the anterior pituitary, are located in the neurosecretory preoptic area (Herget et al., 2014), a well characterized region densely populated by various neuropeptidergic cell types within the aHyp/ORR. In contrast, the functions of the Bsx-dependent clusters expressing *crhb*, *crhbp* and *uts1* in the “PTv,” PRR and TelSep region, respectively, are poorly understood. *uts1* was the only gene for which we found TelSep expression to be Bsx-dependent, providing a first description of a Bsx function in the telencephalon. However, we also found several other genes encoding neuropeptidergic precursors to be expressed in this region, suggesting that the TelSep might act as a neuromodulatory center in zebrafish.

Even though a function in feeding regulation has been suggested for many of the analyzed neuropeptides, they are known to exert multiple functions beyond energy homeostasis, ranging from stress response behavior to the regulation of sleep and arousal. While so far phenotypes of *Bsx* knock-out animals, such as high body fat and reduced locomotion (Sakkou et al., 2007), were discussed only in the light of Bsx as a regulator of *Agrp* and *Npy* expression, alternative explanations should be considered given the breadth of neuropeptides affected.

While Bsx in mouse has been shown to directly bind to the promoters of the *Agrp* and *Npy* genes, we do not know whether this is the case in zebrafish for *agrp, npy*, or any of the other genes for which we found expression to depend on Bsx. It might well be that expression of some of the affected genes in the expected areas was not detected due to a failure of precursors or neurons to migrate or survive in *bsx* mutants. Such roles have recently been described for the Otp transcription factor, which shall be used as an example to illustrate the highly combinatorial action of transcription factors in hypothalamus development. In *Otp* knock-out mice, *Trh*, *Crh*, *Avp*, and *Oxt* and *Sst* expression is lost in specific subregions of the aHyp (Acampora et al., 1999). In the ARC of *Otp* mutant mice, *Agrp*, *Npy* and *Sst* expression is absent while *Ghrh* and *Pomc* expression is normal (Acampora et al., 1999; Lee et al., 2018). A careful re-examination of *Otp* mutant mice revealed that all *Ghrh* expressing cells in the bHyp which originate in alar territories, but migrate to the VMH region, are absent upon Otp loss, while the intrinsically basal *Ghrh* expression in the ARC is unaffected (Morales-Delgado et al., 2014). Unfortunately, we could not analyze *ghrh* expression in *bsx* mutant zebrafish since we could not detect any signal at the embryonic stages analyzed when using a *ghrh* probe.

Since serotonin, Vip and Trh have been implicated in lactation in mammals (Kato et al., 1978; Crowley, 2014), the defects in these systems might contribute to the nursing defects which were described for *Bsx* mutant female mice (McArthur and Ohtoshi, 2007). The hypothalamic nitric oxide system has been linked to ovarian cyclicity and the onset of puberty (Chachlaki et al., 2017) and, notably, *BSX* was identified as a locus for age at menarche in GWAS (Elks et al., 2010; Demerath et al., 2013). Moreover, our data might help to reevaluate endocrine features of patients suffering from Jacobsen syndrome, a disease in which chromosome 11 deletions which encompass the *BSX* locus, were implicated (Haghi et al., 2004; Coldren et al., 2009).

## Materials and Methods

### Zebrafish Strains, Maintenance and Heat Shock Treatment

Zebrafish embryos of the ABTL strain were obtained through natural breeding, incubated in constant darkness at 28°C, and staged according to Kimmel et al. (1995). The following transgenic or mutant lines were used: *Tg(hsp70l:otpa-IRES-gfp-caax)^m1178^* (generated by Heiko Löhr, Driever Lab), *bsx*^m1376^ (Schredelseker and Driever, 2018), *otpa*^m866^ (Ryu et al., 2007), *otpb*^sa115^ (Fernandes et al., 2012), *nkx2.1^m1355^*, *nkx2.4a^m1354^*, *nkx2.4b^m1353^* (this study). All mutant embryos were generated by incrossing heterozygous parent animals. Heat shock was performed by keeping petri dishes with zebrafish embryos in a 39°C water bath for 1 h. Heat shock transgenic embryos were identified through GFP signal by brightfield fluorescent microscopy. All experiments were carried out in accordance with the German Animal Welfare Act.

### Generation of the Heat Shock-Inducible *otpa* Overexpressing Line

The open reading frame of the *otpa* gene (NCBI RefSeq NM_001128703.1) was PCR-amplified from a *pCS2-otpa* plasmid (Ryu et al., 2007) as a template with the following primers containing attB1/2 sites (in bold):

5′**GGGGACAAGTTTGTACAAAAAAGCAGGCT**CGATGC TTTCGCATGCCGACCTGCTG3′,

5′**GGGGACCACTTTGTACAAGAAAGCTGGGT**ATTAGG TGAAGCTCATGGACACTGTG3′

(start and stop codons underlined). PCR-products were combined with a *pDONR221* (Kwan et al., 2007) donor vector in a Gateway BP recombination reaction (Thermo Fisher) to generate the middle entry clone *pME-otpa* which was then recombined with a *p5E-hsp70l* and *p3E-IRES-egfp-caax-pA* onto a *pDestTol2CG2* [for all three plasmids see Kwan et al. (2007)] in a Gateway LR reaction (Thermo Fisher). The resulting *hsp70l-otpa-IRES-egfp-caax-pA-CG2* construct containing Tol2 recombination sites was injected into single-cell-staged embryos together with *transposase* mRNA, which was transcribed *in vitro* from *pCS2FA-transposase* (Kwan et al., 2007) using the mMessage mMachine Sp6 Transcription Kit (Thermo Fisher). The *cmlc2:gfp* cassette (CG2) present on the construct allowed easy screening for germline line transgenic animals through GFP signal in the heart.

### Targeted Mutagenesis of *nkx2.1* and *nkx2.4a/b* Alleles

TALEN target sites were identified for Exon 1 of *nkx2.1* (NCBI Gene ID: 81883), *nkx2.4a* (NCBI Gene ID: 562300) or *nkx2.4b* (NCBI Gene ID: 58112) gene using the Mojo Hand design tool, Version 1.5 (Neff et al., 2013). The following TALEN plasmids were assembled using the Golden Gate TALEN and TAL Effector Kit 2.0 onto an RCI-Script-GoldyTALEN backbone (addgene.org), (Cermak et al., 2011): *nkx2.1* left – GGAGTTTCCCAGCTGTC; *nkx2.1* right – GGGCTACTGTAACGGG (binding strand: CCC GTTACAGTAGCCC); *nkx2.4a* left: GGAAACCTCACATCTCC; *nkx2.4a* right – GCAACCTCAGGTGTCCC (binding strand: GGGACACCTGAGGTTGC); *nkx2.4b* left – GAGAAACGGCGCCACGG; *nkx2.4b* right – GCTCGAA CCCGGAGCCG (binding strand: CGGCTCCGGGT TCGAGC). TALEN assembly products were verified by DNA sequencing and restriction digest with BamHI (NEB) and BsaI (NEB). Purified products were used for *in vitro* transcription of mRNA using the mMessage mMachine T3 Transcription Kit (Thermo Fisher Scientific). For *nkx2.4a* or *nkx2.4b* 200-300 pg of each TALEN mRNA were injected into wildtype one-cell stage embryos. Efficiency of TALEN-mediated indel generation was assayed through PCR and restriction enzyme digest on pooled injected embryos 3 dpf. Potential mutant alleles of F1 animals were sequenced and frameshift indels identified. *nkx2.4a^m1354^*, *nkx2.4b^m1353^* double heterozygous F2 animals were raised and identified through genotyping. Since *nkx2.4a* and *nkx2.1* are both located on chromosome 17, we introduced the *nkx2.1* mutation *de novo* in the *nkx2.4a^m1354^*, *nkx2.4b^m1353^* fish. 200–300 pg *nkx2.1* TALEN mRNA was injected into one cell stage embryos from crosses of *nkx2.4a^m1354^*, *nkx2.4b^m1353^* fish crossed with ABTL fish. A new mutant *nkx2.4b^m1353^* allele with a small deletion generating a frameshift was identified by sequencing in F2 animals. Triple heterozygous *nkx2.1^m1355^*, *nkx2.4a^m1354^*, *nkx2.4b^m1353^* animals were genotyped and crossed to generate *nkx2.1, nkx2.4a, nkx2.4b* triple homozygous embryos.

### Genotyping

All breeding adult animals and experimental embryos were genotyped through PCR, followed by restriction digest. Fin or tail biopsies were lysed in 50 mM NaOH for 45 min at 95°C, then neutralized with 1/10 volume of 1M Tris-HCl pH 7.5 before being used as template. Primer sequences, amplicon length, restriction enzymes and length of digestion products are given in Supplementary Table S2.

### Cloning of cDNA Fragments Used as Templates for *in situ* Hybridization

Gene fragments were PCR amplified from cDNA and cloned into pCRII-TOPO (Invitrogen) as has been described (Schredelseker and Driever, 2018) using primer sequences shown in Supplementary Table S1. Plasmids were linearized with restriction enzymes (Supplementary Table S1) and used as template for *in vitro* transcription of digoxigenin (DIG)-labeled probe using T3, T7, or SP6 RNA polymerase [Supplementary Table S1; Schredelseker and Driever (2018)].

### *In situ* Hybridization and Immunohistochemistry

Gene IDs for all antisense probes generated are provided in Table 1. Whole-mount DIG-labeled RNA *in situ* hybridization based on alkaline phosphatase reaction was carried out as previously described (Schredelseker and Driever, 2018). Tyramide signal amplification (TSA) fluorescent *in situ* hybridization (FISH) was carried out as described previously (Ronneberger et al., 2012). For combined FISH/fluorescent immunohistochemistry (FIHC), after detection of a single DIG-labeled probe through TSA reaction using Alexa Fluor 488, peroxidase activity was inactivated through extensive washing in TNT [100 mM Tris-HCl pH7.5, 150 mM NaCl, 0.5% Tween20] and incubation in 1% H_2_O_2_/TNT for 30 min at room temperature (RT). Embryos were then washed in PBTD [1% DMSO/PBST (0.1% Tween20 in PBS)] and blocked for 2 h in Blocking Solution [5% goat serum (Sigma-Aldrich #G9023), 1% blocking reagent (Roche, #1096176), 1% BSA (Sigma-Aldrich #A6003) in PBTD] at RT. Embryos were incubated with primary rabbit anti 5-HT antibody (Sigma-Aldrich #S5545; 1:200), primary mouse HuC/HuD antibody (16A11, Thermo Fisher Scientific #A-21271; 1:500) or preabsorbed primary rabbit anti-Th antibody (Ryu et al., 2007; 1:500) 1:500 in Blocking Solution over night at 4°C. Embryos were washed all day in PBTD at RT, then incubated with secondary goat anti-rabbit IgG Alexa 488 or 546 antibody (Thermo Fisher Scientific, #A-11008 or #A-11035; 1:1000) and secondary goat anti-mouse Alexa 555 antibody (Thermo Fisher Scientific, #A-21425; 1:1000) in PBTD over night at 4°C. Embryos were washed several times in PBTD and PBST, then transferred to 80% glycerol/PBST and stored at 4°C until imaged. All mutant embryos came from incrosses of heterozygous parent animals and were stained in the same tube and/or wells together with their wildtype siblings, and, after the staining procedure, but before imaging, were genotyped based on tail clip DNA. Heat shock transgenic embryos, identified by GFP signal, were presorted and stained in tubes and/or wells separate from their wildtype siblings, but the staining procedure was performed in parallel, with all solutions being applied at the same time and from the same stock.

**TABLE 1:** Abbreviations, full names, and zfin.org Gene ID of genes in this study.

**Abbreviation**	**Full gene name**	**zfin.org Gene ID**
*agrp*	*agouti related neuropeptide*	ZDB-GENE-040817-1
*avp*	*arginine vasopressin*	ZDB-GENE-030407-2
*bsx*	*brain-specific homeobox*	ZDB-GENE-040628-4
*cart4*	*cocaine- and amphetamine-regulated transcript 4*	ZDB-GENE-060503-863
*crh*	*corticotropin releasing hormone b*	ZDB-GENE-041114-75
*crhbp*	*corticotropin releasing hormone binding protein*	ZDB-GENE-040801-196
*dlx5a*	*distal-less homeobox 5a*	ZDB-GENE-990415-49
*emx2*	*empty spiracles homeobox 2*	ZDB-GENE-990415-54
*fezf2*	*FEZ family zinc finger 2*	ZDB-GENE-001103-3
*foxb1a*	*forkhead box B1a*	ZDB-GENE-990616-47
*galn*	*galanin/GMAP prepropeptide*	ZDB-GENE-111117-2
*ghrh*	*growth hormone releasing hormone*	ZDB-GENE-070426-1
*hcrt*	*hypocretin (orexin) neuropeptide precursor*	ZDB-GENE-040324-1
*hdc*	*histidine decarboxylase*	ZDB-GENE-080102-5
*isl1*	*ISL LIM homeobox 1*	ZDB-GENE-980526-112
*lef1*	*lymphoid enhancer-binding factor 1*	ZDB-GENE-990714-26
*lhx5*	*LIM homeobox 5*	ZDB-GENE-980526-484
*lhx6*	*LIM homeobox 6*	ZDB-GENE-041025-1
*lhx9*	*LIM homeobox 9*	ZDB-GENE-050417-210
*nkx2.1*	*NK2 homeobox 1*	ZDB-GENE-010404-1
*nkx2.4a*	*NK2 homeobox 4a*	ZDB-GENE-030131-6336
*nkx2.4b*	*NK2 homeobox 4b*	ZDB-GENE-000830-1
*nmu*	*neuromedin U*	ZDB-GENE-041001-111
*nos1*	*nitric oxide synthase 1 (neuronal)*	ZDB-GENE-001101-1
*npb*	*neuropeptide B*	ZDB-GENE-040107-40
*npvf*	*neuropeptide VF precursor*	ZDB-GENE-070424-226
*npy*	*neuropeptide y*	ZDB-GENE-980526-438
*nts*	*neurotensin*	ZDB-GENE-091204-433
*nr5a1a*	*nuclear receptor subfamily 5, group A, member 1a*	ZDB-GENE-010504-1
*nr5a2*	*nuclear receptor subfamily 5, group A, member 2*	ZDB-GENE-990415-79
*otpa*	*orthopedia homeobox a*	ZDB-GENE-070216-1
*otpb*	*orthopedia homeobox b*	ZDB-GENE-990708-7
*pax6a*	*paired box 6a*	ZDB-GENE-990415-200
*pax7a*	*paired box 7a*	ZDB-GENE-990415-201
*pdyn*	*prodynorphin*	ZDB-GENE-060417-1
*penka*	*proenkephalin a*	ZDB-GENE-030729-31
*pmch*	*pro-melanin-concentrating hormone*	ZDB-GENE-041210-150
*pmchl*	*pro-melanin-concentrating hormone, like*	ZDB-GENE-030131-7863
*pomca*	*proopiomelanocortin a*	ZDB-GENE-030513-2
*shha*	*sonic hedgehog signaling molecule a*	ZDB-GENE-980526-166
*sst1.1*	*somatostatin 1, tandem duplicate 1*	ZDB-GENE-030131-4743
*th*	*tyrosine hydroxylase*	ZDB-GENE-990621-5
*th2*	*tyrosine hydroxylase 2*	ZDB-GENE-050201-1
*trh*	*thyrotropin-releasing hormone*	ZDB-GENE-020930-1
*uts1*	*urotensin 1*	ZDB-GENE-041014-348
*vip*	*vasoactive intestinal peptide*	ZDB-GENE-080204-3
*vmat2 = slc18a2*	*vesicular monoamine transporter 2 = solute carrier family 18 member 2*	ZDB-GENE-080514-1

**All genes for which antisense RNA probes were used during in situ hybridization. More detail is given in Supplementary Table S1.**

For all previously unpublished probes we performed *in silico* alignment analysis using the BLASTN discontiguous megablast algorithm on the *Danio rerio* nucleotide collection (taxid: 7955) with default parameters and for most probe sequences found no hits except the specific transcript which we aimed to target. There are, however, three exceptions. Our *nos1* probe has some similarity to a *nos2a* transcript (XM_005165296.4). However, *nos2a* was found not to be expressed in the larval zebrafish until 5 dpf (Thisse and Thisse, 2005). For *nr5a1a* we cannot exclude hybridization of our probe to *nr5a1b* (NM_212834.1) or *nr5a2* (NM_001313729.1). Since we used *nr5a1a* staining only to demonstrate that forebrain patterning is normal, we concluded potential hybridization to those other genes to be rather inconsequential for our statement. Our *vmat2* probe also has some similarity to a *vmat1* transcript (XM_021478484.1), which, however, is not expressed in the larval zebrafish brain (Puttonen et al., 2017). We also found the expression patterns generated by all *in situ* hybridization probe sequences cloned in this study to match the expression patterns of the same gene as has been published previously (for references see Supplementary Table S1).

### Imaging and Figure Preparation

Alkaline phosphatase blue *in situ* hybridization stained embryos were mounted for brightfield microscopy in 80% glycerol, 20% PBST, 1 mM EDTA. Images were obtained using an AxioCam CC1 on an AxioPlan2 microscope with a PLAN-NEOFLUAR 20×/0.5 or 10×/0.3 objective and DIC optics using the AxioVs40 Software (all Zeiss). From image stacks (1 μm step size), minimum intensity projections of *z*-planes as indicated in the figure legends were generated using ImageJ. Embryos stained by fluorescent *in situ* hybridization and immunohistochemistry were recorded using a Zeiss LSM 510 or LSM 880. All figures were assembled using Adobe Photoshop CS4 or CS6. When linear adjustment of levels was made, histograms were clipped to the same values for experimental and control images.

## Data Availability Statement

All datasets generated for this study are included in the article/Supplementary Material.

## Ethics Statement

The animal study was reviewed and approved by Regierungspräsidium Freiburg, Freiburg, Germany.

## Author Contributions

TS conceptualized and designed the study and performed the experiments, assembled the figures, and wrote the first draft of the manuscript. FV generated the *nkx2.1* and *nkx2.4a/b* mutant lines. RD communicated unpublished data and suggested hypothalamic markers. WD contributed to design, supervision and editing, and provided project administration and funding acquisition.

## Conflict of Interest

The authors declare that the research was conducted in the absence of any commercial or financial relationships that could be construed as a potential conflict of interest.

## Abbreviations

5-HT: 5-hydroxytryptamine/serotonin
ABas: anterobasal area
AC: anterior commissure
aHyp: alar hypothalamus
ARC: arcuate nucleus
bHyp: basal hypothalamus
CSF-c: cerebrospinal fluid-contacting
DC: diencephalic cluster
dpf: days post fertilization
GWAS: genome-wide association study
Hpf: hours post fertilization
LRR: lateral recess region
Mam: mamillary region
OC: optic commissure
ORR: optic recess region
PBas: posterobasal area
PC: posterior commissure
Pit: pituitary
PM: perimamillary domain
PRM: periretromamillary domain
PRR: posterior recess region
“PTv”, posterior tuberculum: ventral part
PVN: paraventricular nucleus
RM: retromamillary domain
RTu: retrotuberal domain
RTuD, retrotuberal domain: dorsal part
RTuI, retrotuberal domain: intermediate part
RTuV, retrotuberal domain: ventral part
TALEN: transcription activator-like effector nuclease
TCPT: tract of the commissure of the posterior tuberculum
TelSep: telencephalic septum
Tub: tuberal region
TuD, tuberal domain: dorsal part
TuI, tuberal domain: intermediate part
TuV, tuberal domain: ventral part
VMH: ventromedial hypothalamic nucleus region.
