# Supplementary material for: Bsx Is Essential for Differentiation of Multiple Neuromodulatory Cell Populations in the Secondary Prosencephalon
Source: Front Neurosci. 2020 Jun 3;14:525. doi: 10.3389/fnins.2020.00525 (PMC7290237; doi:10.3389/fnins.2020.00525)
Supplement: Supplementary file 1 [file Data_Sheet_1.pdf]

Bsx Is Essential for Differentiation of Multiple Neuromodulatory Cell Populations in the Secondary Prosencephalon

Theresa Schredelseker, Florian Veit, Richard I. Dorsky and Wolfgang Driever

Frontiers in Neuroscience

Gene name	Reference / Primer Sequences	Linearization Enzyme	Polymerase
agrp	received from M. Hammerschmidt Lab<br>fwd: 5’-GAGCACTACAGTGATACATC-3’<br>rev: 5’-ACTTCTCTGTGGATTCTCTG-3’<br>see also Song et al., 2003	BamHI	T7
avp	Tessmar-Raible et al., 2007	EcoRI	SP6
bsx	Schredelseker and Driever, 2018	BamHI	T7
cart4	fwd: 5’-CAAAGCATTCAGCACCATGGAG-3’<br>rev: 5’-TTTCAGTCTCAAGCGTTGCTGTC-3’<br>see also Nishio et al., 2012	NotI	SP6
crhb	Löhr et al., 2009	NotI	SP6
crhbp	fwd: 5’-GCGAACCTACTGACGTTATCAGC-3’<br>rev: 5’-TTCTGACCACAGTGTTATCACAGC-3’<br>see also Thisse et al., 2004	NotI	SP6
dlx5a	Schredelseker and Driever, 2020	NotI	SP6
emx2	Morita et al., 1995	XhoI	T7
fezf2	fwd: 5’-ATGTCCTGCCCGAGACTAGA-3’<br>rev: 5’-GGGTACTGAGGTGTGGGAAA-3’<br>see also Thisse et al., 2005	SpeI	T7
foxb1a	Thisse et al., 2001	EcoRI	T7
galn	fwd: 5’-GCAGAAGAGGCGGGAATATTACC-3’<br>rev: 5’-CTGATCTCTTCTGATGTGAGAGAGGAC-3’<br>see also Podlasz et al., 2012	NotI	SP6
ghrh	fwd: 5’-TGGCTGATTATAACATGGGTGAG-3’<br>rev: 5’-AACCTGGACCAGAAATCTTTGATC-3’	XhoI	SP6
hcrt	received from M. Hammerschmidt Lab;<br>see also Faraco et al., 2006	NotI	SP6
hdc	Yokogawa et al., 2007	EcoRV	SP6
isl1	Tokumoto et al., 1995	XbaI	T3
lef1	Schredelseker and Driever, 2020	NotI	SP6
lhx5	Peng and Westerfield, 2006	SpeI	T7
lhx6	Schredelseker and Driever, 2020	NotI	T3
lhx9	Schredelseker and Driever, 2020	BamHI	T7
nkx2.1	Schredelseker and Driever, 2020	SpeI	T7
nkx2.2a	Schredelseker and Driever, 2020	BamHI	T7
nmu	fwd: 5’-GAGGAACAGCAATCAATGTGAACG-3’<br>rev: 5’-GGTTTGGTGGCACTGATTTTTCAG-3’<br>see also Chiu et al., 2016	BamHI	T7
nos1	fwd: 5’-GTTTGATGACCTCTGGAGAAAGGAC-3’<br>rev: 5’-GATGGTAATAGCTGATCTGAGGTTTCC-3’<br>see also Bradley et al., 2010	NotI	SP6
npb	fwd: 5’-CTGGTGTTCGTAGCTGTTTCC-3’<br>rev: 5’-CAGAAACACGTCTGCTTTGC-3’<br>see also Thisse et al., 2001	NotI	SP6

npvf	fwd: 5'-TGAAGTTACGGCTCTCAGATTGC-3'<br>rev: 5'-CAACAAAACATCTCGACCCATTGC-3'<br>see also Lee et al., 2017	BamHI	T7
npy	received from M. Hammerschmidt Lab;<br>see also Jeong et al., 2018	KpnI	SP6
nr5a1a	fwd: 5'-AAGTTTGGCCCAATGTACAAGC-3'<br>rev: 5'-CCTTGAAGAAGACACAGCTTCTCG-3'<br>see also Muthu et al., 2016	BamHI	T7
nr5a2	Liu et al., 1997	BamHI	T7
nts	fwd: 5'-GTGCCTCCTCTGACATCATACAG-3'<br>rev: 5'TGATGTGAGGAGACTTCTCAGAACC-3'<br>see also Levitas-Djerbi et al., 2015	BamHI	T7
otpa	Schredelseker and Driever, 2020	BamHI	T7
pax6	Krauss et al., 1991	BamHI	T7
pax7a	Seo et al., 1998	BamHI	T7
pdyn	fwd: 5'-ACCACAGGAATCACTGTCGATG-3'<br>rev: 5'-TCGTTTTGGACCGAATTTGCG-3'<br>see also Appelbaum et al., 2010	BamHI	T7
penka	fwd: 5'-GGTGGACTGTGGCTTTGAG-3'<br>rev: 5'-TCCTTCCTCCCAAGTGCG-3'<br>see also Thisse et al., 2004	NotI	SP6
pmch	fwd: 5'-AGCTAGGTTCTGCAACCATCA-3'<br>rev: 5'-CATACATTTCTGCTCGTCATGTT-3'<br>see also Zhang et al., 2010	NotI	SP6
pmchl	fwd: 5'-GATCAAGAATGAAGCTTTCTGCTGG-3'<br>rev: 5'-GTCTTCCCAGAAGTCCTACACC-3'<br>see also Zhang et al., 2010	BamHI	T7
pomca	Herzog et al., 2003	HindIII	T7
shha	Ekker et al., 1995	HindIII	T7
sst1.1	Devos et al., 2002	SalI	SP6
th	Holzschuh et al., 2001	XhoI	T3
trh	Löhr et al., 2009	BamHI	T7
uts1	Wolf and Ryu, 2013	NotI	SP6
vip	Wolf and Ryu, 2013	SacI	T7
vmat2	fwd: 5'-CGACTGTCCCAAAGCAGATG-3'<br>rev: 5' ACTTCCATACACCGACACGT-3'<br>see also Puttonen et al., 2017	XhoI	SP6

Table S1: All genes for which DIG-labeled RNA antisense probes were used for whole-mount *in situ* hybridization. Second column lists reference for all probes which have been published previously and primer sequences for cDNA fragments which were amplified and cloned during this study as is described in the Materials and Methods section. When both primer sequences and a reference is given, we compared the expression pattern of our probe to previously published other probes detecting the samke gene and found the expression patterns to match. Full gene names and zfin.org Gene IDs are given in Table 1.

Allele	fwd primer seq (5'-3')	rev primer seq (5'-3')	PCR Product Size in bp (WT)	Restriction Enzyme	Digest Product Sizes in bp (WT)	Reference
bsx m1376	ATTGCAAAAGGAATGCAGATG	ATTGTCGTCCAGCGTGTATCT	506	XhoI	114, 392	(1)
nkx2.1 m1355	CTTCGAGCTCCCACTCAAAC	TGGCACGTGTAACGTTTAGC	733	NcoI	381, 448	this study
nkx2.4a m1354	ACTGAAAACGTTCGGTCCAC	AGCAGCGAAAACGAGACAAT	829	MspI	334, 399	this study
nkx2.4b m1353	CTAATTTGCAGCAGCACAGC	TGAGTGCGTAACCGAATGAG	554	RsaI	34, 129, 391	this study
otpa m866	GGTCACAGGGAGGCATTAAA	GATAGTGGGTTTTGGCGAAG	310	Hpy188III	140, 170	(2)
otpb sa115	AGGTCAACGCCAAAGACCAA	GCGATCGGAAACATATTTGA	399	BbvI	25, 374	(2)

Table S2: Primer sequences and restriction enzymes used for genotyping mutant alleles. Lysed tissue from fin or tail biopsies was subjected to PCR using the primers given in the second and third column. Amplicons were digested with restriction enzymes listed in the fifth column. Gel electrophoresis of digested PCR reveals genotypes: presence of undigested PCR products alone (similar size to wildtype fragment or 520 bp and 34 bp in the case of *nkx2.4b*) indicates homozygous animal, presence of digestion products alone indicates wildtype animal, presence of both indicates heterozygous animal. References: (1) - Schredelseker and Driever, 2018; (2) - Fernandes et al., 2013.

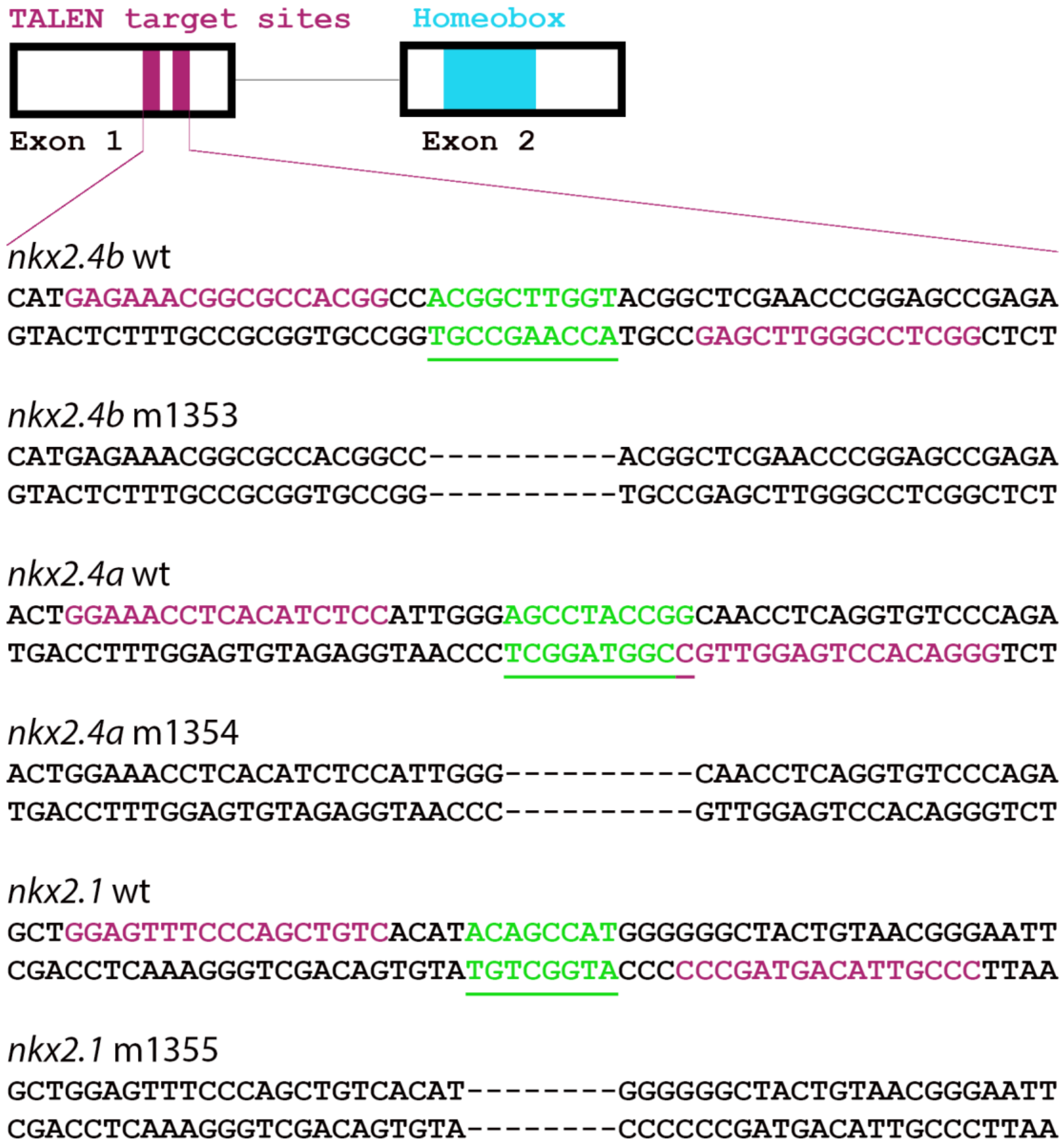

Figure S1: Generation and characterization of *nkx2.1* and *nkx2.4a/b* mutants. Mutant alleles for *nkx2.4b*, *nkx2.4a* and *nkx2.1* were generated by TALEN mutagenesis as described in the Materials and Methods section. For all three genes, coding sequence on Exon 1 was targeted which led to small deletions, frameshifts and premature STOP codons N-terminal to the DNA binding homeodomain (blue) which is encoded on Exon 2 in all three genes. TALEN target sites are shown in purple. Small deletions are shown in green or underlined. All deletions affect a restriction enzyme recognition site allowing PCR and restriction digest-based genotyping as described in Materials and Methods section and Table S2.

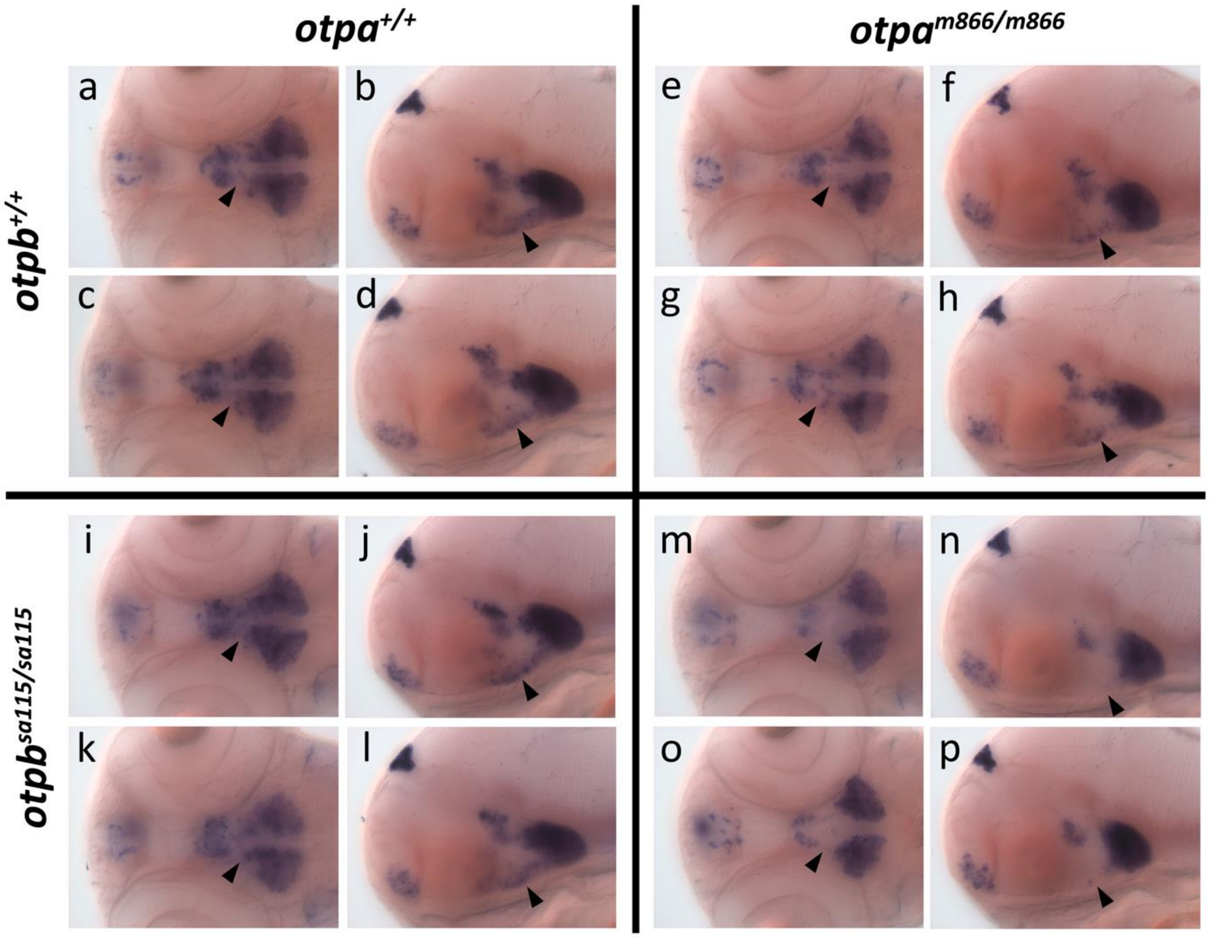

Figure S2: *bsx* expression in *otpa* and *otpb* single and double mutants. Dorsal (a,c,e,g,i,k,m,o) or lateral views (b,d,f,h,j,l,n,p) on zebrafish embryonic forebrains at 3 dpf after *in situ* hybridization using *bsx* probe. Minimum intensity projections of 80 (a,c,e,g,i,k,m,o) or 60 (b,d,f,h,j,l,n,p) brightfield focal planes (1 µm distance). Arrowheads indicate *bsx* expression in the ABas/ARC region, which is absent in *otpa*/*otpb* double mutants (m-p). Note that panels b,h,j,p represent the same images as are shown in Fig.1 e,f,g,h. Left-right adjacent image pairs show ventral and dorsal views on the same embryos. For each genotype a second representative embryo is shown in the second row of each genotype field.

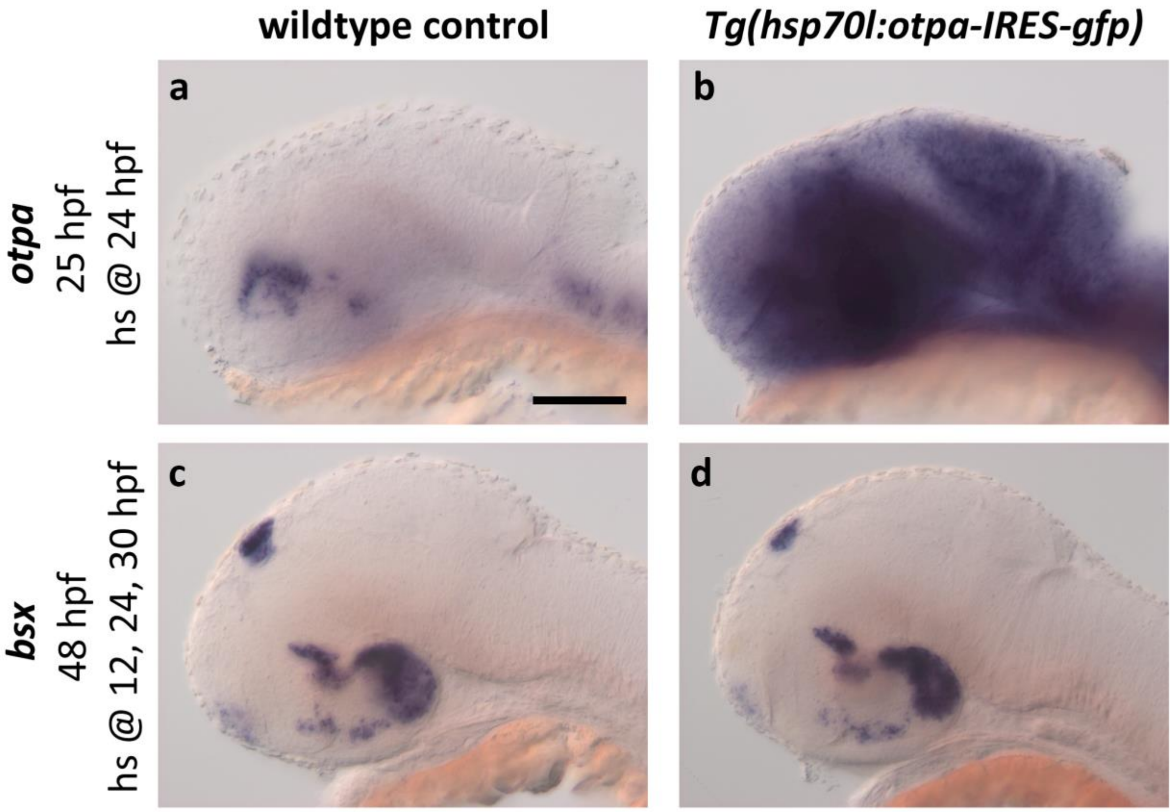

Figure S3: Expression of *otpa* and *bsx* in *otpa* overexpressing transgenic embryos. Lateral view of heads of wildtype (a,c) or transgenic (b,d) embryos 25 hpf (a,b) or 48 hpf (c,d) after *in situ* hybridization using *otpa* (a,b) or *bsx* (c,d) probe. Scale bar 100 µm. Anterior at left.

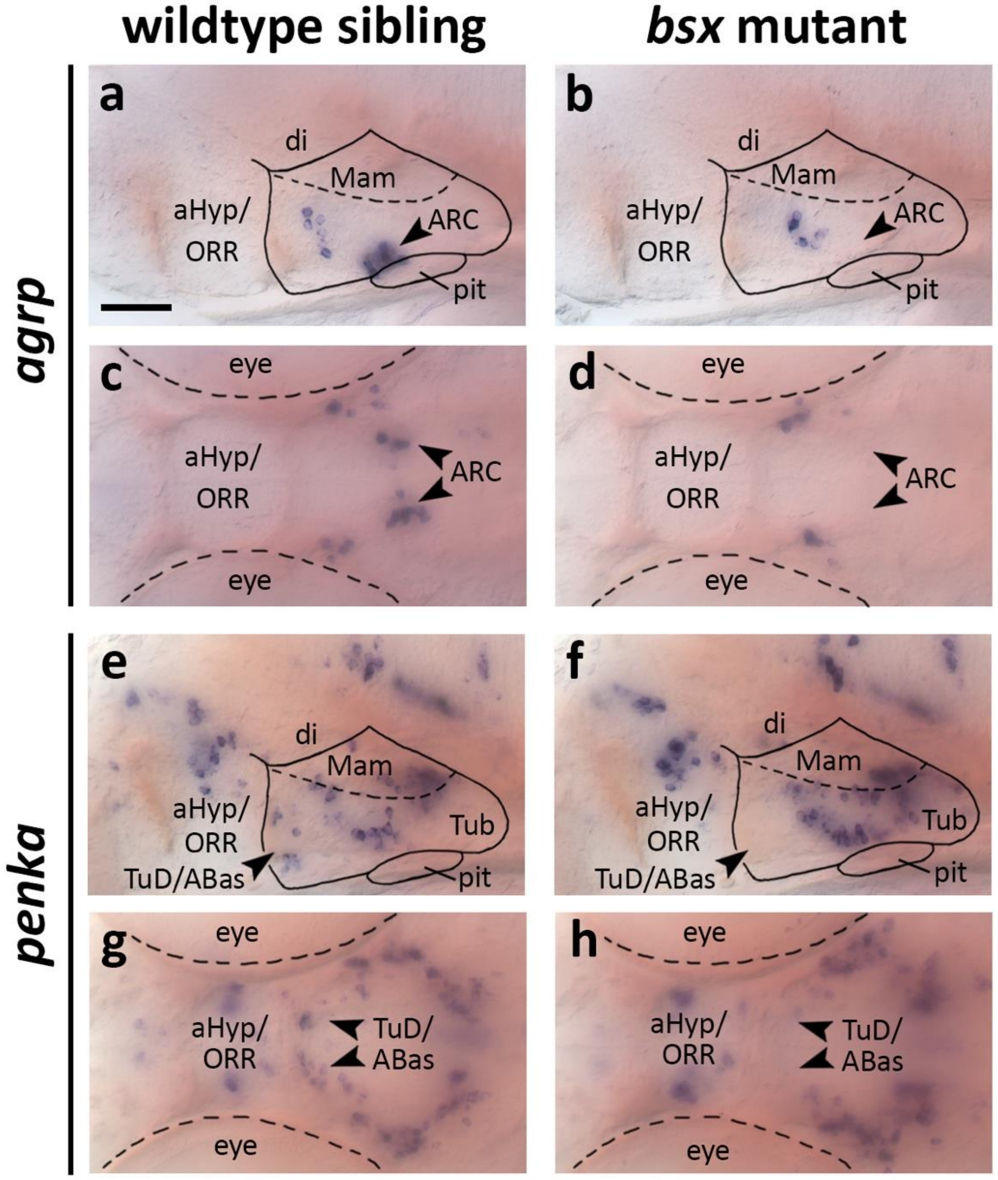

Figure S4: ***agrp*** **and** ***penka*** **expression in wildtype and** ***bsx*** **mutant 4 dpf embryos.** Lateral views (a,b,e,f) and dorsal views (c,d,g,h) of ventral forebrain after *in situ* hybridization using probes as indicated. All images are minimum intensity projections of 40 brightfield focal planes (distance 1 µm). Scale bar 50 µm. n = 11 (a/c); 12 (b/d); 9 (e/g); 4 (f/h). Anterior at left. For abbreviations see list in main text.

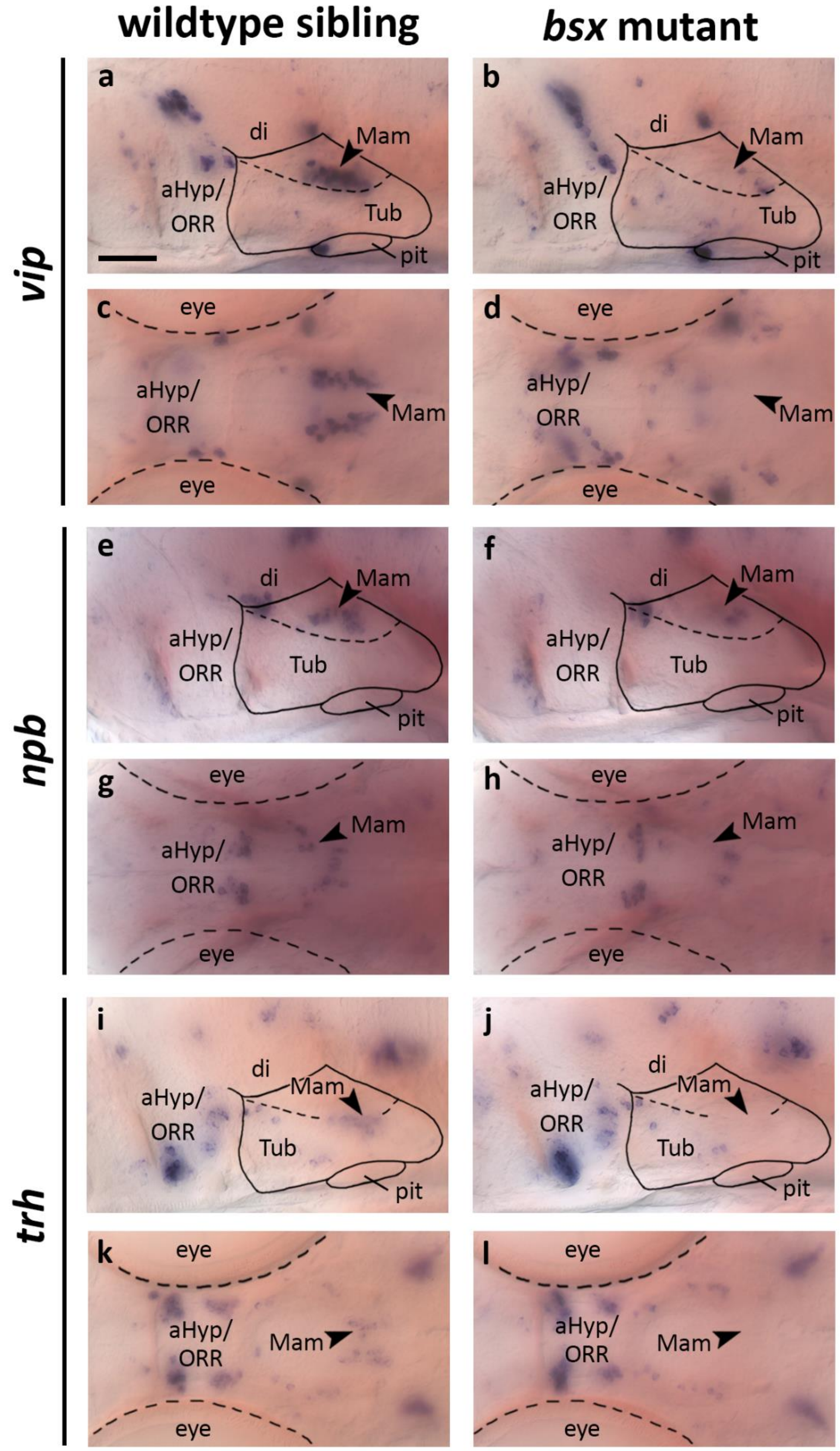

Figure S5: *vip*, *npb* and *trh* expression in wildtype and *bsx* mutant 4 dpf embryos. Lateral views (a,b,e,f,i,j) and dorsal views (c,d,g,h,k,l) of ventral forebrain after *in situ* hybridization using probes as indicated. All images are minimum intensity projections of 40 brightfield focal planes (distance 1 µm). Scale bar 50 µm. n = 4 (a/c); 18 (b/d); 5 (e/g); 8 (f/h, j/l); 2 (i/k). Anterior at left. For abbreviations see list in main text.

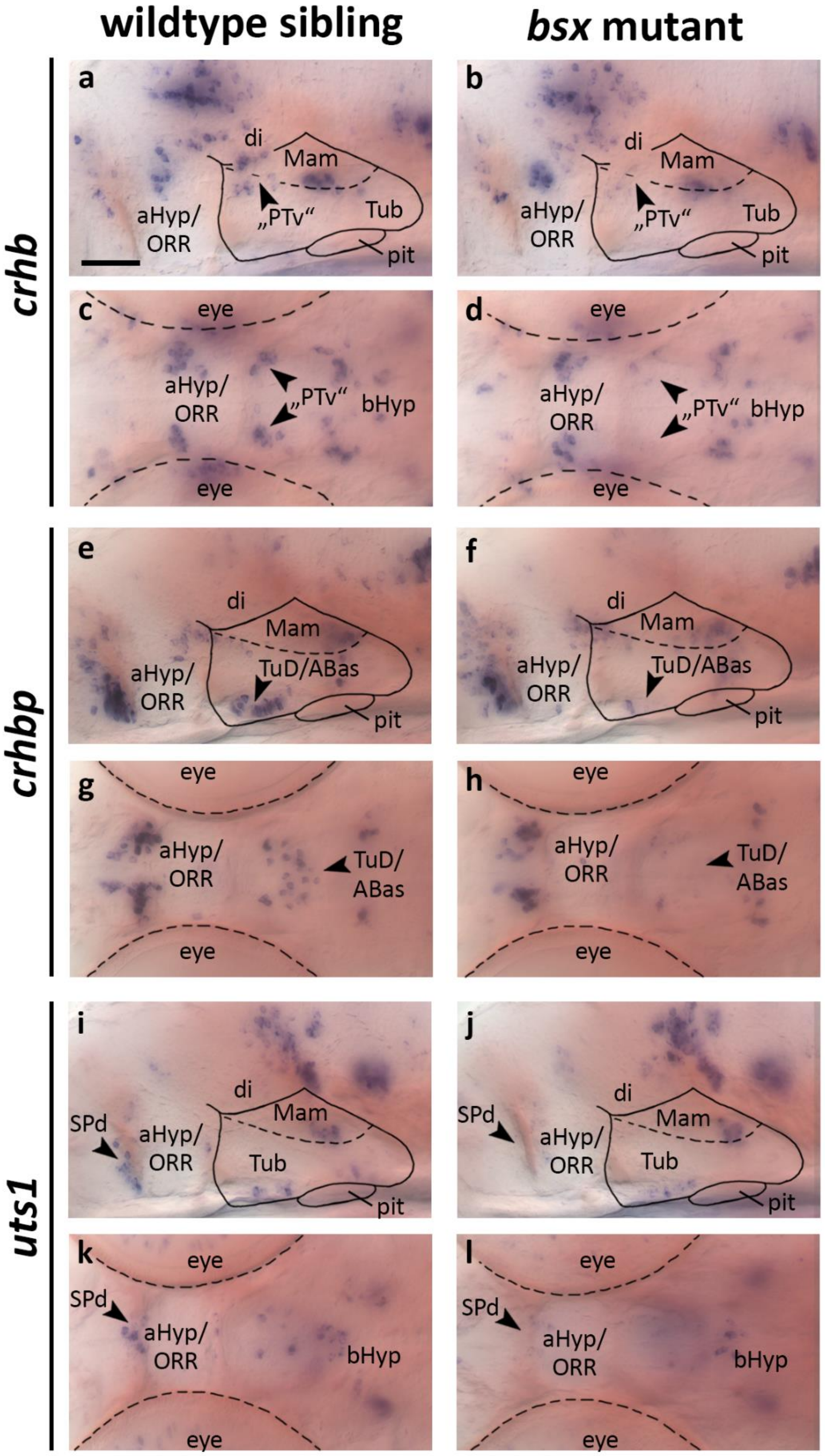

Figure S6: *crhb*, *crhbp* and *uts1* expression in wildtype and *bsx* mutant 4 dpf embryos. Lateral views (a,b,e,f,i,j) and dorsal views (c,d,g,h,k,l) of ventral forebrain after *in situ* hybridization using probes as indicated. All images are minimum intensity projections of 40 brightfield focal planes (distance 1 µm). Scale bar 50 µm. Anterior at left. n = 14 (a/c); 11 (b/d); 5 (e/g); 10 (f/h); 12 (i/k); 3 (j/l). For abbreviations see list in main text.

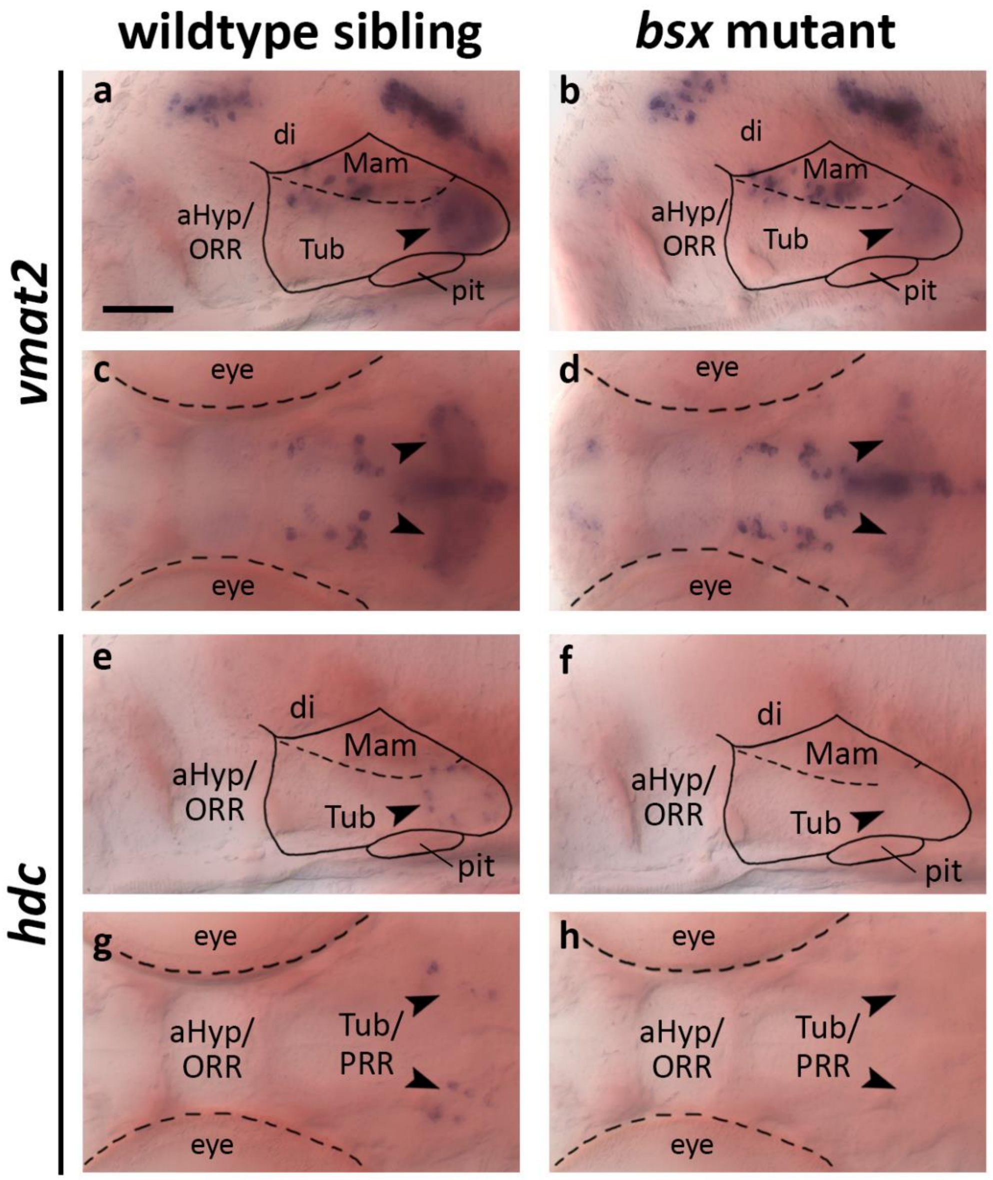

Figure S7: *vmat2* and *hdc* expression in wildtype and *bsx* mutant 4 dpf embryos. Lateral views (a,b,e,f) and dorsal views (c,d,g,h) of ventral forebrain after *in situ* hybridization using probes as indicated. All images are minimum intensity projections of 40 brightfield focal planes (distance 1 µm). Scale bar 50 µm. Anterior at left. n = 6 (a/c); 4 (b/d); 8 (e/g); 9 (f/h). For abbreviations see list in main text.

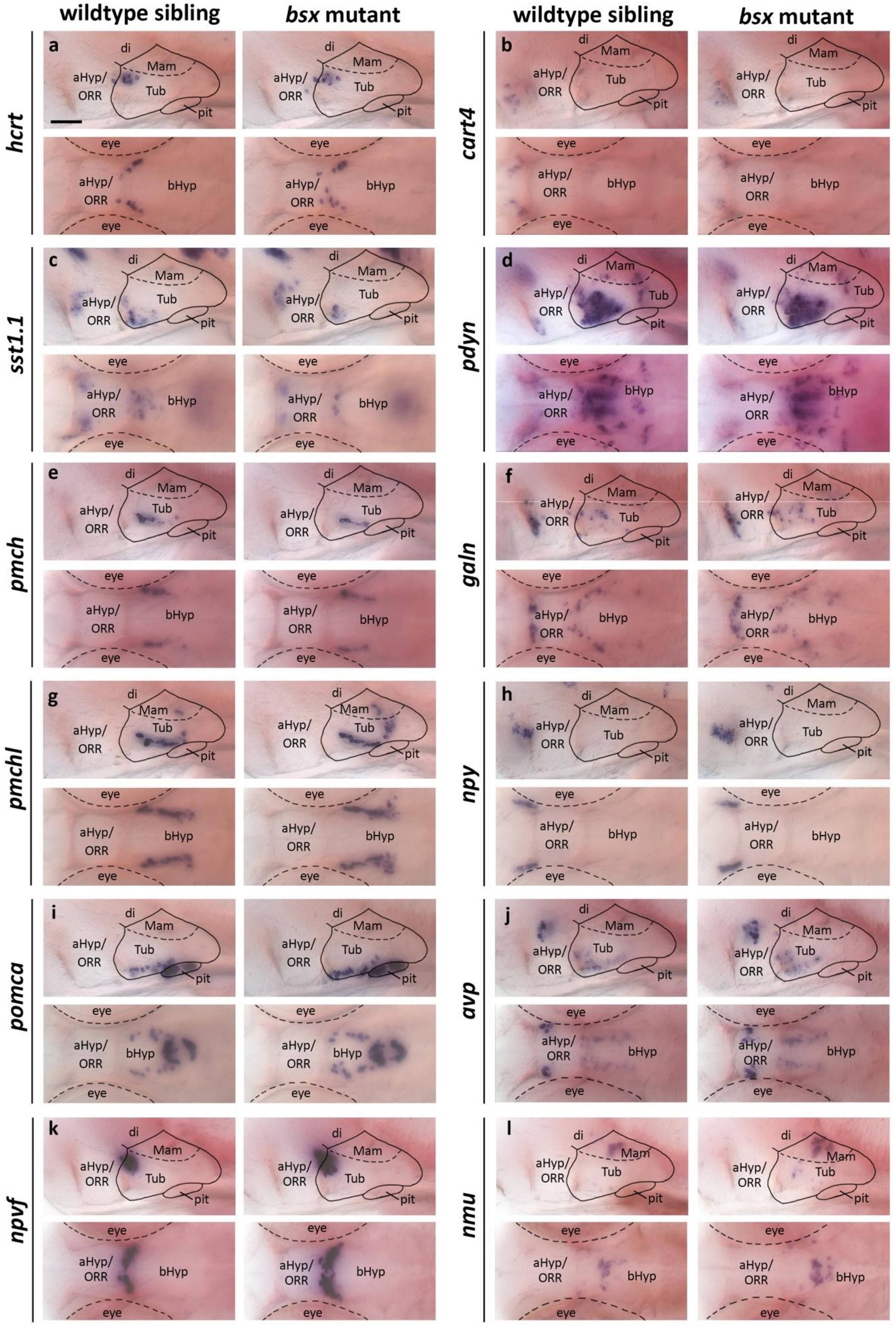

Figure S8: Expression of neuropeptidergic precursor genes in wildtype and *bsx* mutant 3 dpf embryos. Lateral and dorsal views of ventral forebrain after *in situ* hybridization using probes as indicated. All images are minimum intensity projections of 40 brightfield focal planes (distance 1 µm). Scale bar 50 µm. Anterior at left. n (wildtype) = 6 (a,b); 7 (c,l); 3 (e,f); 5 (g,i); 8 (I,k); 16 (d); 14 (j). n (*bsx* mutants) = 11 (a,b); 7 (c,d); 3 (e); 6 (i); 8 (d,h); 5 (f); 9 (j,k); 10 (l). For abbreviations see list in main text.

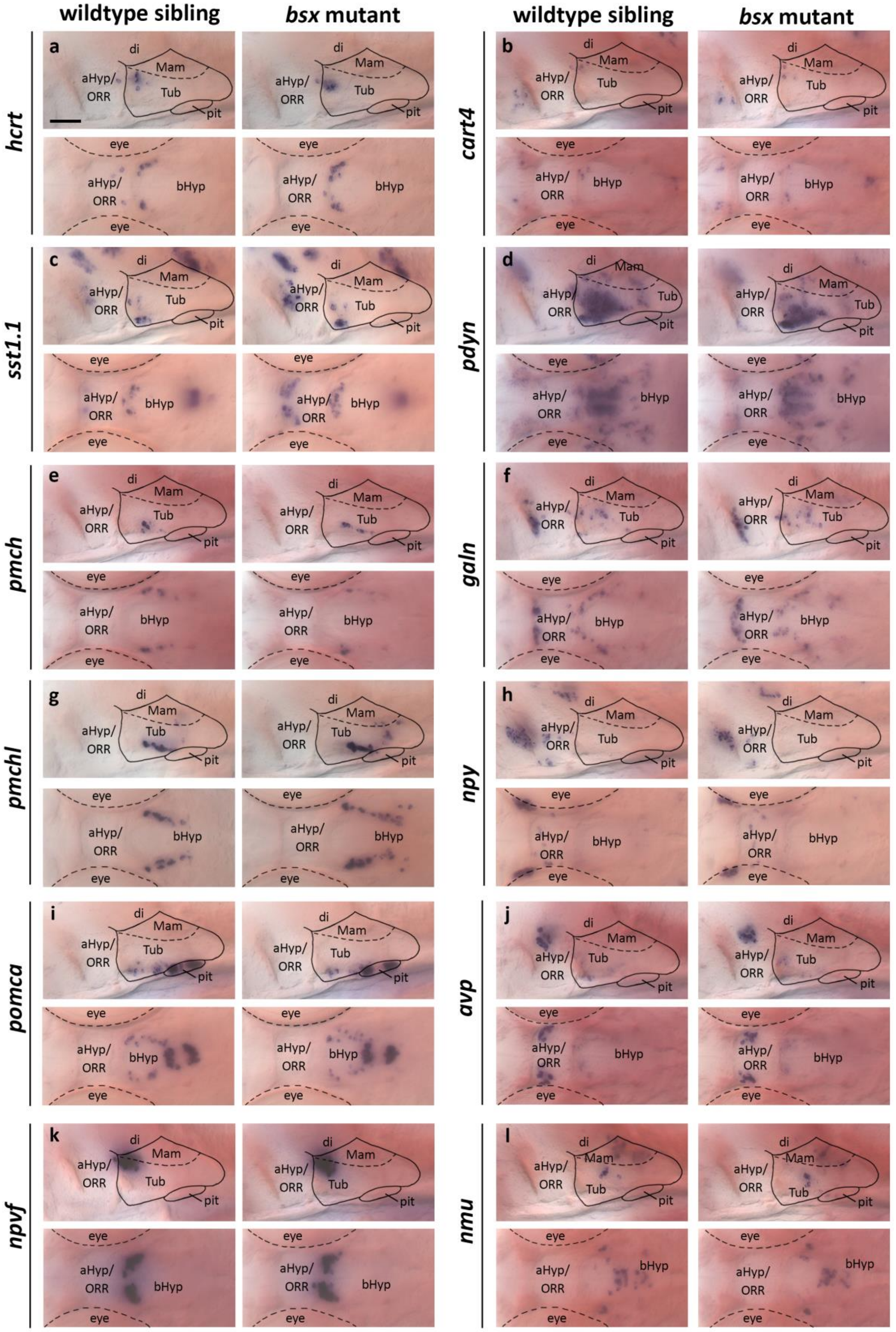

Figure S9: Expression of neuropeptidergic precursor genes in wildtype and *bsx* mutant 4 dpf embryos. Lateral and dorsal views of ventral forebrain after *in situ* hybridization using probes as indicated. All images are minimum intensity projections of 40 brightfield focal planes (distance 1 µm). Scale bar 50 µm. Anterior at left. n (wildtype) = 3 (a,g); 4 (c,h,l); 5 (e); 7 (i); 6(b); 12 (d); 8 (f); 17 (j); 9 (k). n (*bsx* mutants) = 11 (a,j); 4 (c,g); 7 (e,i); 5 (b,h,k); 8 (d); 3 (f); 9 (l). For abbreviations see list in main text.

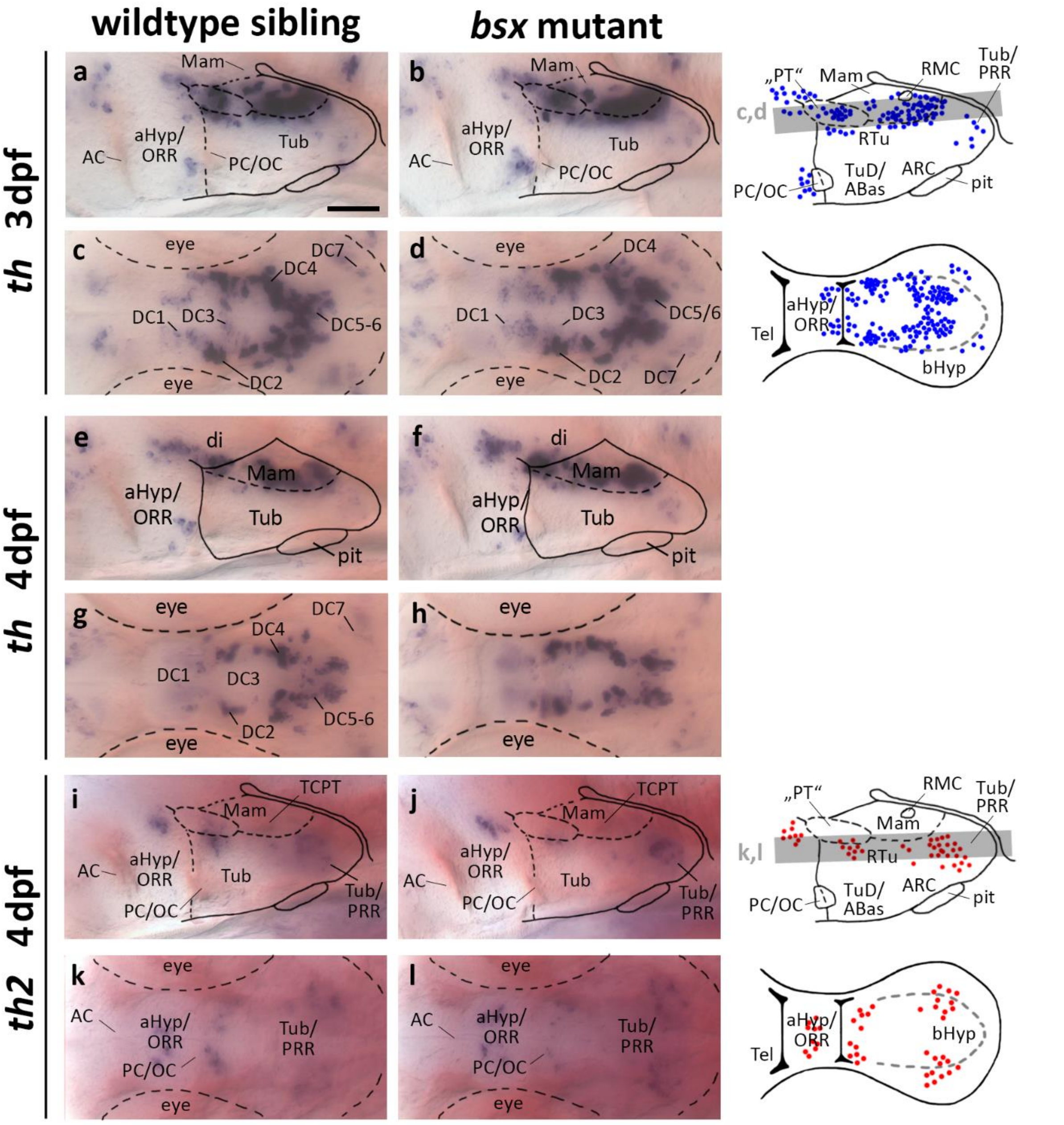

Figure S10: Expression of *th* and *th2* in wildtype and *bsx* mutant 3 or 4 dpf embryos. Lateral (a,b,e,f,i,j) and dorsal (c,d,g,h,k,l) views of ventral forebrain after *in situ* hybridization using probes as indicated. All images are minimum intensity projections of 40 brightfield focal planes (distance 1 µm). Scale bar 50 µm. Anterior at left. n = 6 (a/c); 5 (b/d); 4 (e/g, j/l); 10 (f/h); 14 (i/k). For abbreviations see list in main text.

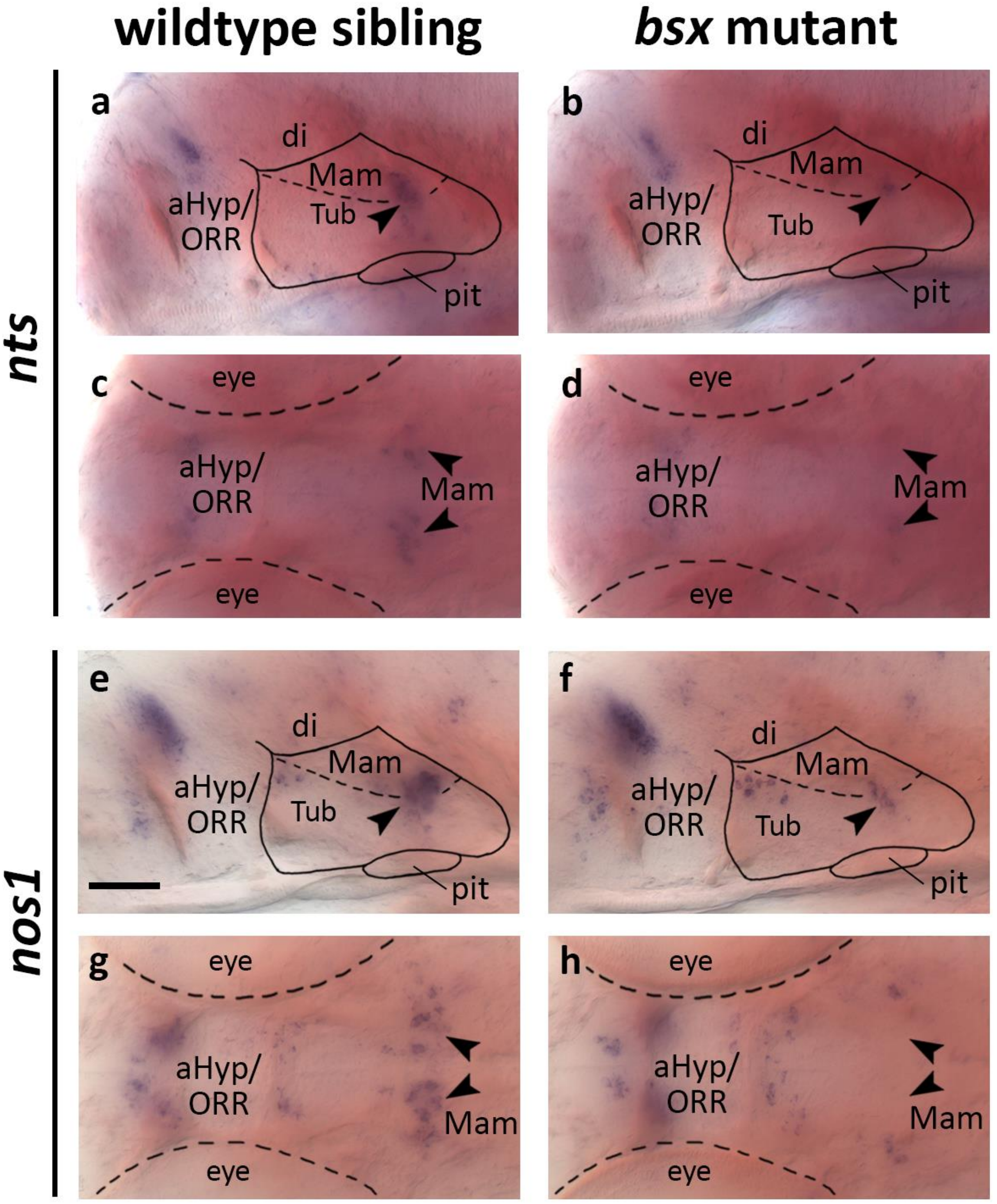

Figure S11: Expression of *nts* and *nos1* in wildtype and *bsx* mutant 4 dpf embryos. Lateral (a,b,e,f) and dorsal (c,d,g,h) views of ventral forebrain after *in situ* hybridization using probes as indicated. All images are minimum intensity projections of 40 brightfield focal planes (distance 1 µm). Scale bar 50 µm. Anterior at left. n = 4 (a/c); 10 (b/d); 6 (e/g); 5 (f/h). For abbreviations see list in main text.

Supplementary References

Appelbaum L, Wang G, Yokogawa T, Skariah GM, Smith SJ, Mourrain P, et al. Circadian and homeostatic regulation of structural synaptic plasticity in hypocretin neurons. *Neuron* (2010) 68**:** 87-98.

Bradley S, Tossell K, Lockley R, McDearmid JR. Nitric Oxide Synthase Regulates Morphogenesis of Zebrafish Spinal Cord Motoneurons. *Journal of Neuroscience* (2010) 30: 16818-16831;

Chiu CN, Rihel J, Lee DA, Singh C, Mosser EA, Chen S, Sapin V, Pham U, Engle J, Niles BJ, Montz CJ, Chakravarthy S, Zimmerman S, Salehi-Ashtiani K, Vidal M, Schier AF, Prober DA.A Zebrafish Genetic Screen Identifies Neuromedin U as a Regulator of Sleep/Wake States. *Neuron* (2016) 89: 842-856.

Devos N, Deflorian G, Biemar F, Bortolussi M, Martial JA, Peers B, et al. Differential expression of two somatostatin genes during zebrafish embryonic development. *Mechanisms of Development* (2002) 115**:** 133-137.

Ekker SC, Ungar AR, Greenstein P, von Kessler DP, Porter JA, Moon RT, et al. Patterning activities of vertebrate hedgehog proteins in the developing eye and brain. *Current Biology* (1995) 5**:** 944-955.

Faraco JH, Appelbaum L, Marin W, Gaus SE, Mourrain P, Mignot E. Regulation of Hypocretin (Orexin) Expression in Embryonic Zebrafish. *Journal of Biological Chemistry* (2006) 281**:** 29753-29761.

Fernandes AM, Beddows E, Filippi A, Driever W. Orthopedia Transcription Factor otpa and otpb Paralogous Genes Function during Dopaminergic and Neuroendocrine Cell Specification in Larval Zebrafish. *PLOS One* (2013) 8**:** e75002.

Herzog W, Zeng X, Lele Z, Sonntag C, Ting J-W, Chang C-Y, et al. Adenohypophysis formation in the zebrafish and its dependence on sonic hedgehog. *Developmental Biology* (2003) 254**:** 36-49.

Holzschuh J, Ryu S, Aberger F, Driever W. Dopamine transporter expression distinguishes dopaminergic neurons from other catecholaminergic neurons in the developing zebrafish embryo. *Mechanisms of Development* (2001) 101**:** 237-243.

Jeong I, Kim E, Kim S, Kim H-K, Lee D-W, Seong JY, et al. mRNA expression and metabolic regulation of npy and agrp1/2 in the zebrafish brain. *Neuroscience Letters* (2018) 668**:** 73-79.

Krauss S, Johansen T, Korzh V, Fjose A. Expression of the zebrafish paired box gene pax[zf-b] during early neurogenesis. *Development* (1991) 113**:** 1193-1206.

Lee DA, Andreev A, Truong TV, Chen A, Hill AJ, Oikonomou G, et al. Genetic and neuronal regulation of sleep by neuropeptide VF. *Elife* (2017) 6.

Levitas-Djerbi T, Yelin-Bekerman L, Lerer-Goldshtein T, Appelbaum L. Hypothalamic leptin-neurotensin-hypocretin neuronal networks in zebrafish. *The Journal of Comparative Neurology* (2015) 523**:** 831-848.

Liu D, Le Drean Y, Ekker M, Xiong F, Hew CL. Teleost FTZ-F1 Homolog and Its Splicing Variant Determine the Expression of the Salmon Gonadotropin IIβ Subunit Gene. *Molecular Endocrinology* (1997) 11**:** 877-890.

Löhr H, Ryu S, Driever W. Zebrafish diencephalic A11-related dopaminergic neurons share a conserved transcriptional network with neuroendocrine cell lineages. *Development* (2009) 136**:** 1007-1017.

Morita T, Nitta H, Kiyama Y, Mori H, Mishina M. Differential expression of two zebrafish emx homeoprotein mRNAs in the developing brain. *Neuroscience Letters* (1995) 198**:** 131-134.

Muthu V, Eachus H, Ellis P, Brown S, Placzek M. Rx3 and Shh direct anisotropic growth and specification in the zebrafish tuberal/anterior hypothalamus. *Development* (2016) 143**:** 2651-2663.

Nishio S-I, Gibert Y, Berekelya L, Bernard L, Brunet F, Guillot E, Le Bail J-C, Sánchez JA, Galzin AM, Triqueneaux G, Laudet V. Fasting Induces CART Down-Regulation in the Zebrafish Nervous System in a Cannabinoid Receptor 1-Dependent Manner. *Molecular Endocrinology* (2012) 26: 1316–1326.

Peng G, Westerfield M. Lhx5 promotes forebrain development and activates transcription of secreted Wnt antagonists. *Development* (2006) 133**:** 3191.

Podlasz P, Sallinen V., Chen Y-C, Kudo H, Fedorowska N, Panula P. Galanin gene expression and effects of its knock-down on the development of the nervous system in larval zebrafish. *The Journal of Comparative Neurology* (2012) 520: 3846-3862.

Puttonen HAJ, Semenova S, Sundvik M, Panula P. Storage of neural histamine and histaminergic neurotransmission is VMAT2 dependent in the zebrafish. *Scientific Reports* (2017) 7**:** 3060.

Schredelseker T, Driever W. Bsx controls pineal complex development. *Development* (2018) 145, dev143677.

Schredelseker T, Driever W. Conserved Genoarchitecture of the Basal Hypothalamus in Zebrafish Embryos. *Frontiers in Neuroanatomy* (2020) 14**:** 3.

Seo H-C, Sætre BO, Håvik B, Ellingsen S, Fjose A. The zebrafish Pax3 and Pax7 homologues are highly conserved, encode multiple isoforms and show dynamic segment-like expression in the developing brain. *Mechanisms of Development* (1998) 70**:** 49-63.

Song Y, Golling G, Thacker TL, Cone, RD. Agouti-related protein (AGRP) is conserved and regulated by metabolic state in the zebrafish, *Danio rerio*. *Endocrine* (2003) **22,** 257–265.

Tessmar-Raible K, Raible F, Christodoulou F, Guy K, Rembold M, Hausen H, et al. Conserved Sensory-Neurosecretory Cell Types in Annelid and Fish Forebrain: Insights into Hypothalamus Evolution. *Cell* (2007) 129**:** 1389-1400.

Thisse B, Pflumio S, Fürthauer M, Loppin B, Heyer V, Degrave A, et al. (2001). Expression of the zebrafish genome during embryogenesis. *ZFIN Direct Data Submission*. Available: https://zfin.org/ZDB-PUB-010810-1T5. Accessed: May 12, 2020.

Thisse B, Thisse, C. (2004) Fast Release Clones: A High Throughput Expression Analysis. *ZFIN Direct Data Submission*. Available: https://zfin.org/ZDB-PUB-040907-1. Accessed: May 12, 2020.

Thisse C, Thisse B. (2005) High Throughput Expression Analysis of ZF-Models Consortium Clones. *ZFIN Direct Data Submission*. Available: https://zfin.org/ZDB-PUB-051025-1. Accessed: May 12, 2020.

Tokumoto M, Gong Z, Tsubokawa T, Hew CL, Uyemura K, Hotta Y, et al. Molecular Heterogeneity among Primary Motoneurons and within Myotomes Revealed by the Differential mRNA Expression of Novel Islet-1 Homologs in Embryonic Zebrafish. *Developmental Biology* (1995) 171**:** 578-589.

Wolf A, Ryu S. Specification of posterior hypothalamic neurons requires coordinated activities of Fezf2, Otp, Sim1a and Foxb1.2. *Development* (2013) 140**:** 1762-1773.

Yokogawa T, Marin W, Faraco J, Pézeron G, Appelbaum L, Zhang J, et al. Characterization of Sleep in Zebrafish and Insomnia in Hypocretin Receptor Mutants. *PLOS Biology* (2007) 5**:** e277.

Zhang C, Song Y, Thompson DA, Madonna MA, Millhauser GL, Toro S, et al. Pineal-specific agouti protein regulates teleost background adaptation. *Proceedings of the National Academy of Sciences of the United States of America* (2010) 107**:** 20164-20171.
